# Derived category of weak chain U-complexes

**DOI:** 10.1016/j.heliyon.2024.e35257

**Published:** 2024-07-29

**Authors:** Fajar Yuliawan, Intan Muchtadi-Alamsyah, Valerian Pratama, Gustina Elfiyanti

**Affiliations:** a Algebra Research Group, Faculty of Mathematics and Natural Sciences, Institut Teknologi Bandung, Indonesia; b Master's Program in Mathematics, Faculty of Mathematics and Natural Sciences, Institut Teknologi Bandung, Indonesia; c Department of Mathematics, Faculty of Science and Technology, Syarif Hidayatullah State Islamic University Jakarta, Indonesia

**Keywords:** 18E30, Derived category, Weak chain U-complex

## Abstract

In this paper, we define the derived category of weak chain U-complexes, and we give a characterization of any weak chain U-complex as an object in the right bounded homotopy category of weak chain U-complexes of projective modules. Then, we show that the homology functor between the category of chain complexes and weak chain U-complexes is a natural isomorphism by constructing an adjoint pair between these two categories.

## 1 Introduction

Let *R* be a ring with identity. A sequence $X={\left(X_{n},d_{n}^{X}\right)}_{n\in Z}$ of *R*-modules and *R*-modules homomorphisms(1)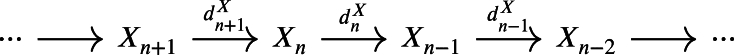 is called a chain complex if $d_{n}^{X}d_{n+1}^{X}\left(X_{n+1}\right)=0$ for every n∈Z. Davvaz and Shabani-Solt modified this definition by replacing the zero submodule of $X_{n-1}$ with any submodule $U_{n-1}$ of $X_{n-1}$. They called the sequence (1) a chain U-complex if it satisfies $d_{n}^{X}d_{n+1}^{X}\left(X_{n+1}\right)\subseteq U_{n-1}^{X}$, and Im(dnX)⊇Un−1X for every n∈Z (see [1, Definition 2.1]). This concept was motivated by a notion of exact sequences of hypergroups introduced by Freni and Sureau in [2]. Since a hypergroup does not always have a zero element, they defined the kernel of a hypergroup homomorphism as the inverse image of U where U is the intersection of all ultra-closed subhypergroups of its codomain.

Recently, some authors continued working on U-exactness. Mahatma and Muchtadi-Alamsyah defined U-projective resolutions and U-extension modules [3]. Baur et al. then computed the U-projective resolution of modules over *kQ* where *Q* is a quiver of type $A_{n}$ and ${\overset{˜}{A}}_{n}$. [4]

In [5] and [6], Elfiyanti et al. showed that the category of chain U-complexes and the homotopy category of chain U-complexes are additive categories. But, the mapping cone M(f) of a morphism f:X→Y of U-complexes defined in [1, Definition 2.2] is not always an object in the category of chain U-complexes because Im(dnM(f)) does not always contain $U_{n-1}^{M(f)}$ (see [7, Example 3]). However, for every n∈Z, we have $d_{n}^{M\left(f\right)}\left(U_{n}^{M\left(f\right)}\right)\subseteq U_{n-1}^{M\left(f\right)}$. We also have that $d_{n}^{X}\left(U_{n}^{X}\right)\subseteq U_{n-1}^{X}$ for any chain U-complex $X={\left(X_{n},U_{n}^{X},d_{n}^{X}\right)}_{n\in Z}$. Motivated by this fact, in [7] Elfiyanti et al. proposed a concept of weak chain U-complexes by replacing the second condition of a chain U-complex *X*, i.e., Im(dnX)⊇Un−1X, with $d_{n}^{X}\left(U_{n}^{X}\right)\subseteq U_{n-1}^{X}$. They used the term “weakly U-complex.” They proved that the weak chain U-complexes category is an additive category, and the homotopy category of weak chain U-complexes is triangulated. It is natural to ask whether we can use their results to construct the derived category of weak chain U-complexes.

The paper is organized as follows. In section 2, we review the category of chain U-complexes in 2.1 and the homotopy category of chain U-complexes in 2.2. We also review the homology of weak chain U-complexes in 2.3. We then review mapping cone in $K_{U}(R)$ and recall that the category $K_{U}(R)$ of chain U-complexes is a triangulated category in 2.4.

In section 3, we construct a weak U-projective resolution of a module *M* by following the argument in [3] and we show that this resolution is unique up to homotopy.

Section 4 introduces a quasi-isomorphism of weak chain U-complexes and studies its properties. Then, we define the derived category $D_{U}(R)$ of chain U-complexes as a localization of the homotopy category of chain U-complexes concerning quasi-isomorphisms of chain U-complexes in 4.1. Finally, we identify an object in the derived category of weak chain U-complex as an object in the right bounded homotopy category of weak chain U-complexes of projective modules in 4.2.

In section 5, we define a functor from the category of chain complexes C(R) to the category of weak chain U-complexes $C_{U}(R)$ in 5.1 and the reverse direction in 5.2. We then show that they form an adjoint pair in 5.3, and we use that fact to show that there is a natural isomorphism between homology functors from C(R) and from $C_{U}(R)$ in 5.4.

**Notation** Unless otherwise specified, we use the following notations throughout this paper: *R* denotes a ring with identity. Chain complexes and their generalizations are over *R*-Mod, the category of *R*-modules. We denote by C(R), U-$C\left(R\right),C_{U}\left(R\right)$ the category of chain complexes, chain U-complexes, and weak chain U-complexes, respectively. We also denote by

• $C_{U}^{+}(R)$ the category of left bounded weak chain U-complexes.
• $C_{U}^{-}(R)$ the category of right bounded weak chain U-complexes.
• $C_{U}^{b}(R)$ the category of bounded weak chain U-complexes.
• $C_{U}^{\emptyset ,+}(R)$ the category of weak chain U-complexes with left bounded homology.
• $C_{U}^{\emptyset ,-}(R)$ the category of weak chain U-complexes with right bounded homology.
• $C_{U}^{\emptyset ,b}(R)$ the category of weak chain U-complexes with bounded homology.
• $C_{U}^{-,b}(R)$ the category of left bounded weak chain U-complexes with bounded homology.
• $C_{U}^{+,b}(R)$ the category of right bounded weak chain U-complexes with bounded homology.

and replacing C with **K** for their respective homotopy category.

## 2 Preliminaries

In this section, we recall some basic concepts we will need in the following sections. For more detail see [1, Section 2], [7, Section 4] and [8, Section 1].

### 2.1 The category of weak chain U-complexes

We start this section by recalling the definition of weak chain U-complex.

Definition 2.1 [7, Section 4] A chain $X={\left(X_{n},U_{n}^{X},d_{n}^{X}\right)}_{n\in Z}$, where $X_{n}$ is an *R*-module, $U_{n}^{X}$ is a submodule of $X_{n}$ and $d_{n}^{X}:X_{n}\to X_{n-1}$ is an *R*-module homomorphism,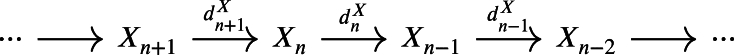 is a **weak chain** U**-complex** if for every n∈Z we have:

1. $d_{n}^{X}d_{n+1}^{X}\left(X_{n+1}\right)\subseteq U_{n-1}^{X}$, and
2. $d_{n}^{X}\left(U_{n}^{X}\right)\subseteq U_{n-1}^{X}$

The morphism of weak chain U-complexes is defined as analogous to the morphism of chain U-complexes.

Definition 2.2 Suppose that $X={\left(X_{n},U_{n}^{X},d_{n}^{X}\right)}_{n\in Z}$ and $Y={\left(Y_{n},U_{n}^{Y},d_{n}^{Y}\right)}_{n\in Z}$ are two weak chain U-complexes. The chain mapping $f={\left(f_{n}:X_{n}\to Y_{n}\right)}_{n\in Z}$ is a **morphism of weak chain** U**-complexes** if $f_{n}d_{n+1}^{X}=d_{n+1}^{Y}f_{n+1}$ and $f_{n}\left(U_{n}^{X}\right)\subseteq U_{n}^{Y}$ for every n∈Z.

The category of weak chain U-complexes can be defined analogously to the category of chain U-complexes.

Definition 2.3 The **category of weak chain** U**-complexes**, denoted by $C_{U}(R)$, is a category whose objects are weak chain U-complexes, the morphisms are the morphisms of weak chain U-complexes, and the composition is the chain mapping composition.

Proposition 2.4 *[7, Proposition 4]* *. The category of weak chain* U *-complexes* $C_{U}(R)$ *is an additive category.*

### 2.2 The homotopy category of weak chain U-complexes

The homotopy relation on the weak chain U-complexes is defined as follows.

Definition 2.5 Suppose that $X={\left(X_{n},U_{n}^{X},d_{n}^{X}\right)}_{n\in Z}$ and $Y={\left(Y_{n},U_{n}^{Y},d_{n}^{Y}\right)}_{n\in Z}$ are two weak chain U-complexes.

1. A morphism of weak chain U-complexes $f={\left(f_{n}:X_{n}\to Y_{n}\right)}_{n\in Z}$ is **nullhomotopic**, written as f∼0, if there is a morphism of weak chain U-complexes $s={\left(s_{n}:X_{n}\to Y_{n+1}\right)}_{n\in Z}$
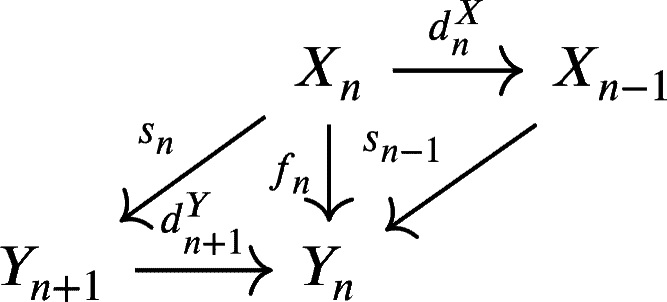
 which satisfies $f_{n}=s_{n-1}d_{n}^{X}+d_{n+1}^{Y}s_{n}$ for every n∈Z.
  We call the morphism $s={\left(s_{n}:X_{n}\to Y_{n+1}\right)}_{n\in Z}$ a **homotopic chain** of weak chain U-complexes.
2. Two morphisms of weak chain U-complexes f,g are **homotopic**, denoted by f∼g, if f−g∼0.
3. A weak chain U-complex *X* is **nullhomotopic** if $1_{X}∼0$.
4. A morphism of weak chain U-complexes $f={\left(f_{n}:X_{n}\to Y_{n}\right)}_{n\in Z}$ is a **chain equivalence** if there is a weak chain U-complex morphism g:Y→X such that $fg∼1_{Y}$ and $gf∼1_{C}$.
5. Two weak chain U-complexes *X* and *Y* are **chain equivalent** if there is a chain equivalence between them.

As in the U-complexes category, the homotopic relation above is also an equivalence relation. The homotopic relation is also closed under the composition of weak chain U-complexes morphism.

Lemma 2.6 *The weak chain* U *-complex homotopy relation is an equivalence relation in* $\mathrm{Mor}(C_{U}(R))$ *.*

Lemma 2.7 *If* f∼g:X→Y *and* $f^{\prime }∼g^{\prime }:Y\to Z$ *, then*

$$f^{\prime }f∼g^{\prime }g:X\to Z$$

The proof is analogous to the proof for chain U-complexes in [1, Lemma 2.9].

Corollary 2.8 *Suppose that X and Y are objects in the category*$C_{U}(R)$*. The collection of all*$I\left(X,Y\right)=\left\{f\in {\mathrm{Hom}}_{C_{U}\left(R\right)}\;\left(X,Y\right)|f∼0\right\}$*forms an ideal on*$\mathrm{Mor}\;\left(C_{U}(R)\right)$*. In other words if*f,g∈I(X,Y)*,*$\alpha \in {\mathrm{Hom}}_{C_{U}\left(R\right)}\;\left(W,X\right)$*and*$\beta \in {\mathrm{Hom}}_{C_{U}\left(R\right)}\;\left(Y,Z\right)$*then*β(f−g)α∈I(W,Z)*.*

The homotopy category of weak chain U-complexes is defined as the quotient of the category of weak chain U-complexes modulo this ideal.

Definition 2.9 The **homotopy category of weak chain** U**-complexes**, denoted by $K_{U}\left(R\right)$, is a category that has the same objects as $C_{U}\left(R\right)$, the morphism is the weak chain U-complex morphisms modulo homotopy, i.e.

$${\mathrm{Hom}}_{K_{U}\left(R\right)}\;\left(X,Y\right)={\mathrm{Hom}}_{C_{U}\left(R\right)}\;\left(X,Y\right)/I\left(X,Y\right)$$

For all x,y∈{∅,b,+,−} we denote by $K_{U}^{x,y}(R)$ the category with objects the same objects as $C_{U}^{x,y}(R)$ and with morphisms being homotopy equivalence classes of morphisms of weak chain U-complexes.

Proposition 2.10 *[7, Proposition 5]* *. The homotopy category of weak chain* U *-complexes,* $K_{U}\left(R\right)$ *, is an additive category.*

### 2.3 Homology of weak chain U-complexes

The homology of weak chain U-complexes is defined slightly different from the homology of chain U-complexes because by the definition of weak chain U-complexes, $U_{n}^{X}$ should be in ${\left(d_{n}^{X}\right)}^{-1}(U_{n-1}^{X})$ for all n∈Z.

Definition 2.11 Suppose ${\left(X_{n},U_{n}^{X},d_{n}^{X}\right)}_{n\in Z}$ is a weak chain U-complex. The *n*-th homology of *X* is the module

Hn(X)=(dnX)−1(Un−1X)UnX+Im(dn+1X)

Recall that for submodules *A* and *B* of *M*, their sum, denoted by A+B, is defined as the submodules of *M* whose elements have the form a+b, with a∈A and b∈B.

Remark 2.12

(i) Since $d_{n}^{X}\circ d_{n+1}^{X}(X_{n+1})\subseteq U_{n-1}^{X}$ and $d_{n}^{X}(U_{n}^{X})\subseteq U_{n-1}^{X}$ by the definition of weak chain *U*-complexes, we have that $U_{n}^{X}$ and Im(dn+1X) are both mapped by $d_{n}$ inside $U_{n-1}^{X}$. Therefore UnX+Im(dn+1X) is a submodule of $d_{n}^{-1}(U_{n-1}^{X})$ which is the preimage of $U_{n-1}^{X}$ under $d_{n}$.
(ii) If *X* is the usual chain complex, i.e., $U_{n}=0$ for all n∈Z, then the *n*-th homology of *X* as a weak chain *U*-complex is the same as the usual definition of homology, that is
  Hn(X)=Ker(dnX)Im(dn+1X).
  This justifies the terminology in the definition above.

As in the chain of U-complexes, the following properties also hold in weak chain U-complexes.

Lemma 2.13 *Suppose* ${\left(X_{n},U_{n}^{X},d_{n}^{X}\right)}_{n\in Z}$ *and* ${\left(Y_{n},U_{n}^{Y},d_{n}^{Y}\right)}_{n\in Z}$ *are two weak chain* U *-complexes and* $f={\left(f_{n}:X_{n}\to Y_{n}\right)}_{n\in Z}$ *is a morphism of weak chain* U *-complexes, then:*

1. $f_{n}\left({\left(d_{n}^{X}\right)}^{-1}\left(U_{n-1}^{X}\right)\right)\subseteq {\left(d_{n}^{Y}\right)}^{-1}\left(U_{n-1}^{Y}\right)$ *.*
2. fn(UnX+Im(dn+1X))⊆UnY+Im(dn+1Y) *.*

Proof Consider the following commutative diagram:
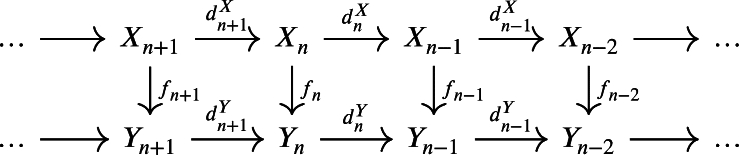

1. For any $y_{n}\in f_{n}\left({\left(d_{n}^{X}\right)}^{-1}\left(U_{n-1}^{X}\right)\right)$, $y_{n}=f_{n}\left(x_{n}\right)$ for an $x_{n}\in {\left(d_{n}^{X}\right)}^{-1}\left(U_{n-1}^{X}\right)$. This means $d_{n}^{X}\left(x_{n}\right)\in U_{n-1}^{X}$, consequently $f_{n-1}d_{n-1}^{X}\left(x_{n}\right)\in U_{n-1}^{Y}$. Since the above diagram commutes, then $f_{n-1}d_{n}^{X}\left(x_{n}\right)=$ $d_{n}^{Y}f_{n}\left(x_{n}\right)\in U_{n-1}^{Y}$. Thus, we can conclude that $y_{n}=f_{n}\left(x_{n}\right)$ $\in {\left(d_{n}^{Y}\right)}^{-1}\left(U_{n-1}^{Y}\right)$.
2. Take any xn∈UnX+Im(dn+1X), then $x_{n}=u_{n}+v_{n}$ for a $u_{n}\in U_{n}^{X}$ and vn∈Im(dn+1X). Therefore $f_{n}\left(x_{n}\right)=f_{n}\left(u_{n}\right)+f_{n}\left(v_{n}\right)$. Because vn∈Im(dn+1X) then $v_{n}=d_{n+1}^{X}\left(x_{n+1}\right)$ for an $x_{n+1}\in X_{n+1}$. Thus fn(vn)=fn(dn+1X(xn+1))=dn+1Y(fn+1(xn+1))∈Im(dn+1Y). We conclude that fn(UnX+Im(dn+1X))⊆UnY+Im(dn+1Y). □

Theorem 2.14 *Suppose that* ${\left(X_{n},U_{n}^{X},d_{n}^{X}\right)}_{n\in Z}$ *and* ${\left(Y_{n},U_{n}^{Y},d_{n}^{Y}\right)}_{n\in Z}$ *are two weak chain* U *-complexes. If* $f={\left(f_{n}:X_{n}\to Y_{n}\right)}_{n\in Z}$ *is a morphism of weak chain* U *-complexes, then f induces an R-module homomorphism* $H\left(f\right)={\left(H_{n}\left(f\right)\right)}_{n\in Z}$ *with:*

Hn(f):Hn(X)⟶Hn(Y)xn+(UnX+Imdn+1X)↦fn(xn)+(UnY+Imdn+1Y)

Proof First, we will prove that the above mapping is well-defined. Suppose xn+(UnX+Imdn+1X)=xn′+(UnX+Imdn+1X), then xn−xn′∈UnX+Imdn+1X. Based on Lemma 2.13, we get fn(xn)−fn(xn′)=fn(xn−yn)∈UnY+Imdn+1Y. Thus, fn(xn)+(UnY+Imdn+1Y)=fn(xn′)+(UnY+Imdn+1Y). So, we conclude that $H_{n}(f)$ is well-defined. Thus, *f* induces H(f), which is an *R*-module homomorphism. □

We also observe the relationship between homotopy and homology given by the following theorem.

Theorem 2.15 *If* f,g:X→Y *are two morphisms of weak chain* U *-complexes and* f∼g *then* $H_{n}\left(f\right)=H_{n}\left(g\right)$ *.*

Proof Let f∼g:X→Y, then there is $s={\left(s_{n}:X_{n}\to Y_{n+1}\right)}_{n\in Z}$
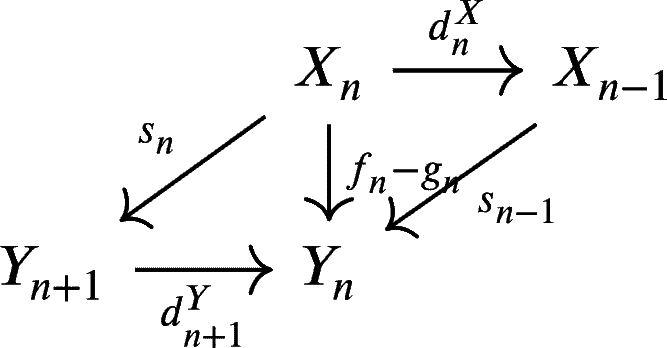
 which satisfies $s_{n}\left(U_{n}^{X}\right)\subseteq U_{n+1}^{Y}$ and $f_{n}-g_{n}=s_{n-1}d_{n}^{X}+d_{n+1}^{Y}s_{n}$ for every n∈Z. Take any an=xn+(UnX+Imdn+1X)∈Hn(X). We will prove (Hn(f−g))(an)∈UnY+Imdn+1Y.

Hn(f−g)(an)=(fn−gn)(xn)+(UnY+Imdn+1Y)=(sn−1dnX+dn+1Ysn)(xn)+(UnY+Imdn+1Y)=sn−1dnX(xn)+dn+1Ysn(xn)+(UnY+Imdn+1Y)

Since $x_{n}\in d_{n}^{X-1}\left(U_{n-1}^{X}\right)$, then $d_{n}^{X}\left(x_{n}\right)\in U_{n-1}^{X}$. Consequently, $s_{n-1}d_{n}^{X}\left(x_{n}\right)\in U_{n}^{Y}$. Thus, we have that Hn(f−g)(an)∈UnY+Imdn+1Y. We now conclude that $H_{n}\left(f\right)=H_{n}\left(g\right)$. □

Corollary 2.16 *Nullhomotopic morphisms induce the zero morphism on homology.*

Definition 2.17 A morphism of chain complex f:X→Y is a quasi-isomorphism if it induces an isomorphism $H_{n}(f):H_{n}(X)\to H_{n}(Y)$ for all n∈Z. We also define a quasi-isomorphism similarly for a morphism of weak chain U-complex (by using homology of weak chain U-complexes instead).

### 2.4 Mapping cone and triangulated property

Before we define the mapping cone of a morphism of chain U-complexes, we first define the translation functor. Suppose that $X={\left(X_{n},U_{n}^{X},d_{n}^{X}\right)}_{n\in Z}$ and $Y={\left(Y_{n},U_{n}^{Y},d_{n}^{Y}\right)}_{n\in Z}$ are two objects in $C_{U}(R)$ and f:X→Y is a morphism in $C_{U}(R)$. Let

$$\Sigma X={\left(\Sigma X_{n},U_{n}^{\Sigma X},d_{n}^{\Sigma X}\right)}_{n\in Z}$$

with $\Sigma X_{n}=X_{n-1}$, $U_{n}^{\Sigma X}=U_{n-1}^{X}$, and $d_{n}^{\Sigma X}=-d_{n-1}^{X}$. Note that

$$d_{n}^{\Sigma X}d_{n+1}^{\Sigma X}\left(\Sigma X_{n+1}\right)=-d_{n-1}^{X}\left(-d_{n}^{X}\left(X_{n}\right)\right)=d_{n-1}^{X}d_{n}^{X}\left(X_{n}\right)\subseteq U_{n-2}^{X}=U_{n-1}^{\Sigma X}$$

and

$$d_{n}^{\Sigma X}\left(U_{n}^{\Sigma X}\right)=-d_{n-1}^{X}\left(U_{n-1}^{X}\right)\subseteq U_{n-2}^{X}=U_{n-1}^{\Sigma X}.$$

Define $\Sigma f={\left(\Sigma f_{n}\right)}_{n\in Z}$ with $\Sigma f_{n}=f_{n-1}$ for every n∈Z, then obviously Σ*f* is also a morphism in $C_{U}(R)$. Hence, the translation functor in $C_{U}\left(R\right)$ can be defined analogously to the translation functor on C(R) as follows.

Definition 2.18 [7, Definition 8] In the category of weak chain U-complexes $C_{U}\left(R\right)$, the **translation functor** $\Sigma :C_{U}(R)⟶C_{U}(R)$ is defined by the shift of any object and any morphism in $C_{U}\left(R\right)$ one degree to the left. In other words, for every object $X={\left(X_{n},U_{n}^{X},d_{n}^{X}\right)}_{n\in Z}$ object in $C_{U}\left(R\right)$, define

$$\Sigma X={\left(\Sigma X_{n},U_{n}^{\Sigma X},d_{n}^{\Sigma X}\right)}_{n\in Z}$$

with $\Sigma X_{n}=X_{n-1},\;U_{n}^{\Sigma X}=U_{n-1}^{X}$, and $d_{n}^{\Sigma X}=-d_{n-1}^{X}$. Then, for any f:X→Y morphism in $C_{U}\left(R\right)$, define

$$\Sigma f={\left(\Sigma f_{n}\right)}_{n\in Z}\text{ with }\Sigma f_{n}=f_{n-1}$$

The additive property of the above functor is derived from the additive property of the translation functor on C(R). Further if f∼g then we have Σf∼Σg, so the translation functor Σ is also well-defined in $K_{U}\left(R\right)$.

Definition 2.19 Suppose that f:X⟶Y is a morphism in $C_{U}\left(R\right)$. The **mapping cone** of *f* is an object $M\left(f\right)={\left(M{\left(f\right)}_{n},d_{n}^{M\left(f\right)},U_{n}^{M\left(f\right)}\right)}_{n\in Z}$ with $M{\left(f\right)}_{n}=X_{n-1}⊕Y_{n}$, $d_{n}^{M\left(f\right)}=\left(\begin{array}{cc} -d_{n-1}^{X}\; & 0\\ f_{n-1}\; & d_{n}^{Y} \end{array}\right):X_{n-1}⊕Y_{n}⟶X_{n-2}⊕Y_{n-1}$ and $U_{n}^{M\left(f\right)}=U_{n-1}^{X}⊕U_{n}^{Y}$.

By [7, Lemma 2], M(f) is an object in $K_{U}\left(R\right)$.

Example 2.20 Here are some examples of mapping cones.

1. Suppose X,Y are any objects in $C_{U}\left(R\right)$. If f:X→0 is a morphism in $C_{U}\left(R\right)$ then M(f)=ΣX. On the other hand, if g:0→Y is a morphism in $C_{U}\left(R\right)$ then M(g)=Y.
2. Suppose $X=\left(X_{n},U_{n}^{X}d_{n}^{X}\right)$ is a complex in $C_{U}\left(R\right)$, then
  $$M\left(1_{X}\right)=\left(X_{n-1}⊕X_{n},U_{n-1}^{X}⊕U_{n}^{X},d_{n}^{M\left(1_{X}\right)}\right)$$
  with
  $$d_{n}^{M\left(1_{X}\right)}=\left(\begin{array}{cc} -d_{n-1}^{X}\; & 0\\ 1_{X_{n-1}}\; & d_{n}^{X} \end{array}\right):X_{n-1}⊕X_{n}⟶X_{n-2}⊕X_{n-1}.$$

Lemma 2.21 *[7, Lemma 3]* *Identity morphism on mapping cone* $M\left(1_{X}\right)$ *is nullhomotopic.*

The mapping cone M(f) relates to *X* and *Y* through the following canonical mapping.

Lemma 2.22 *[7, Lemma 4]**Suppose*f:X⟶Y*is a morphism in*$C_{U}\left(R\right)$*, the canonical morphisms in*${\;\;C}_{U}(R)$*are*

$$\alpha \left(f\right):Y⟶M\left(f\right)\;\text{with}\;\alpha {\left(f\right)}_{n}=\left(\begin{array}{c} 0\\ 1_{Y_{n}} \end{array}\right)$$

*and*

$$\alpha \left(f\right):M\left(f\right)⟶\Sigma X\;\text{with}\;\alpha {\left(f\right)}_{n}=\left(\begin{array}{cc} 1_{X_{n-1}}\; & 0 \end{array}\right).$$

Now, we will see the triangulated structure of the category $K_{U}(R)$ by using the mapping cones.

Definition 2.23 A **triangle** in the homotopy category $K_{U}(R)$ is a 6-tuple (X,Y,Z,f:X→Y,g:Y→Z,h:Z→ΣX) of objects and morphisms in $K_{U}(R)$
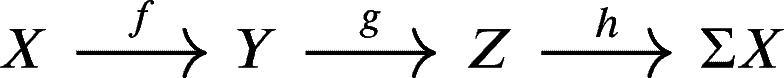

A **morphism of triangles** (X,Y,Z,f,g,h) and $\left(X^{\prime },Y^{\prime },Z^{\prime },f^{\prime },g^{\prime },h^{\prime }\right)$ is a triple morphism (r,s,t) such that the following diagram is commutative in $K_{U}(R)$.
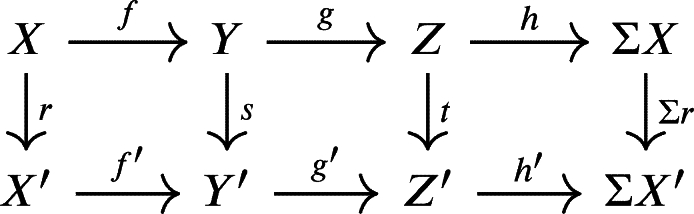

The triple (r,s,t) above is a **triangle isomorphism** if each r,s,t is an isomorphism in $K_{U}(R)$.

Definition 2.24 [7, Definition 9] A **distinguished triangle** in $K_{U}(R)$ is a triangle which is isomorphic (in $K_{U}(R)$) to a triangle of the form(2)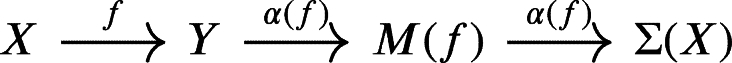 The triangle in the equation (2) above is also called a **standard triangle**.

In the next theorem, we see that the homotopy category of weak chain U-complexes is a triangulated category. Recall that a triangulated category is defined as follows.

Definition 2.25 [8, Definition 3.1] A triangulated category is an additive category T together with an additive automorphism Σ, the translation or shift functor, and a collection of distinguished triangles satisfying the following axioms:

TR0 Any triangle isomorphic to a distinguished triangle is again a distinguished triangle.
TR1 For every object *X* in T, the triangle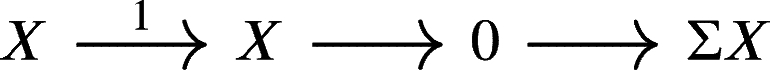 is a distinguished triangle.
TR2 For every morphism f:X⟶Y in T there is a distinguished triangle of the form
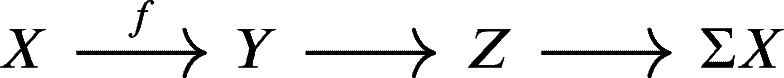
TR3 If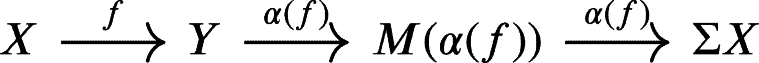 is a distinguished triangle, then the following rotated triangle is also distinguished.
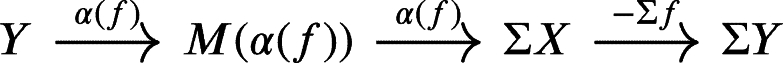
TR4 Given distinguished triangles  and  then, each commutative diagram
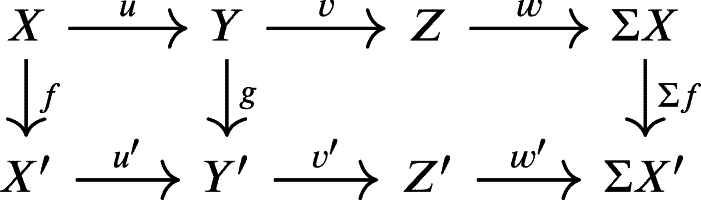
 can be completed to a morphism of triangles (but not necessarily uniquely).
TR5 (Octahedral axiom) Given the following distinguished triangles
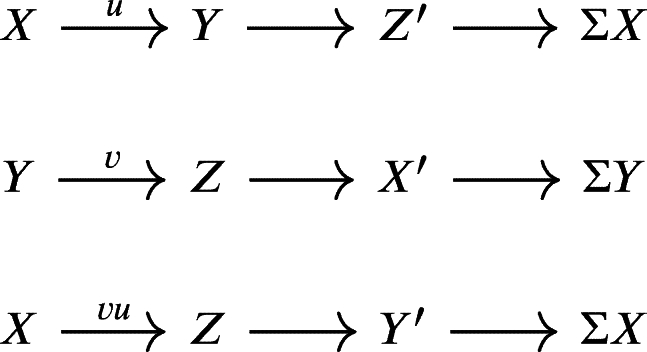
 then, there exists a distinguished triangle  making the following diagram commutative
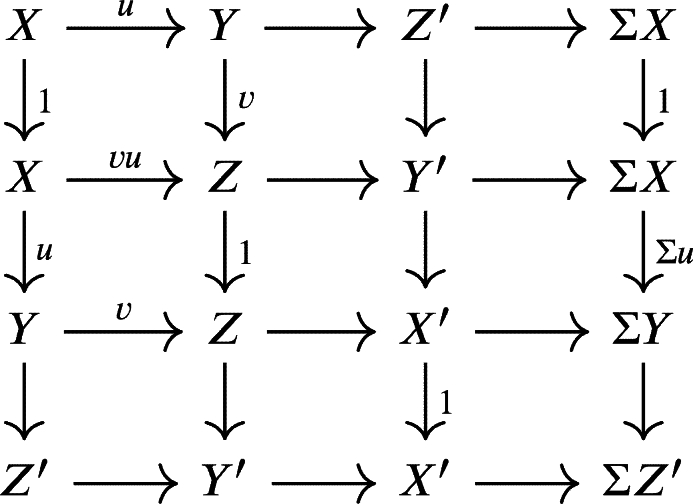

The homotopy category $K_{U}\left(R\right)$ together with the translation functor $\Sigma :K_{U}\left(R\right)\to K_{U}\left(R\right)$ and the class of distinguished triangles (2) form a triangulated category.

Theorem 2.26 *[7, Theorem 1]* *The homotopy category* $K_{U}\left(R\right)$ *of weak chain* U *-complexes is a triangulated category.*

## 3 Weak U-projective resolutions

Following the argument of [3], we will construct a weak U-projective resolution of an *R*-module *M*. We modify the notion of exactness in the usual projective resolution with weak U-exactness as follows. Given *R*-modules A,B,C with $U_{B}$ and $U_{C}$ submodules of *B* and *C*, respectively, a sequence
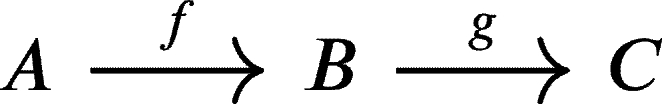
 with *f* and *g* are *R*-module homomorphisms is **weak** U**-exact at** *B* if Imf+UB=g−1(UC).

For an *R*-module *M* and a submodule *U* of *M*, let us consider an exact sequence
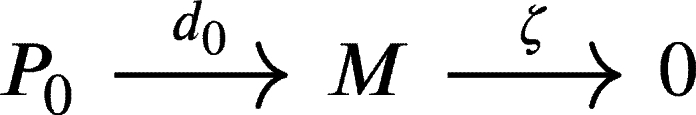
 with $P_{0}$ a projective module. Here, $d_{0}$ will denote the canonical projection. Now, we define $U_{0}=d_{0}^{-1}(U)$ and consider the weak U-exact sequence at $P_{0}$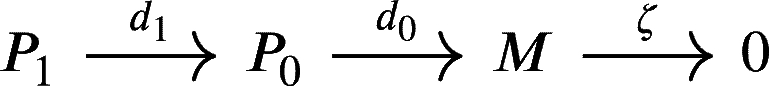 by appending the previous exact sequence by a projective module $P_{1}$. To guarantee the existence, we can take $P_{1}$, a projective module, and a projection $\epsilon_{1}:P_{1}\to \mathrm{Ker}\;d_{0}\cap U_{0}$. Now, notice that

$$d_{0}(d_{1}(P_{1})+U_{0})=d_{0}d_{1}(P_{1})+d_{0}(U_{0})=d_{0}(U_{0})=U$$

Hence, we have Imd1+U0=d0−1(U).

As the next step, we define $U_{1}:=d_{1}^{-1}(U_{0})$ and $P_{2}$ a projective module, and a projection $\epsilon_{2}:P_{2}\to \mathrm{Ker}\;d_{1}\cap U_{1}$. Now, notice that Imd2⊆U1, and hence, we have Imd2+U1=U1=d1−1(U0).

In a more general case, let $P_{n},P_{n-1},\ldots$ be projective modules with each successive projective module $P_{n}$ is chosen with a projection $\epsilon_{n}:P_{n}\to \mathrm{Ker}\;d_{n}\cap U_{n}$ and a weak U-exact sequence
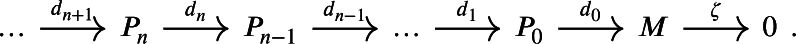


For every *n*, let $U_{n}=d_{n}^{-1}(U_{n-1})$. It follows similarly that we have Imdn+Un−1=dn−1−1(Un−2), so the sequence is weak U-exact.

If U=0, then $U_{0}=\mathrm{Ker}\;d_{0}$, $P_{1}$ is a projective module along with a projection $\epsilon_{1}:P_{1}\to \mathrm{Ker}\;d_{0}$ and $U_{1}=d_{1}^{-1}(\mathrm{Ker}\;d_{0})=P_{1}$. Then $P_{2}$ is a projective module along with a projection $\epsilon_{2}:P_{2}\to \mathrm{Ker}\;d_{1}$ and $U_{2}=d_{2}^{-1}(P_{1})=P_{2}$. More generally, $P_{n}$ is the projective cover of $\mathrm{Ker}\;d_{n-1}$ a projective module along with a projection $\epsilon_{n}:P_{n}\to \mathrm{Ker}\;d_{n-1}$ and $U_{n}=d_{n}^{-1}(P_{n-1})=P_{n}$. Then we get

Imdn+Un−1=Un−1=dn−1−1(Pn)=dn−1−1(Un).

We then conclude that every *R*-module *M* has a weak U-projective resolution by this construction.

The sequence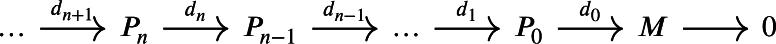 constructed as above is called a **weak** U**-projective resolution of** *M***,** and we denote as $P_{•}:P_{•}(U_{•})\overset{d_{•}}{\to }M(U)$. Now we will show that, by a similar construction as in [3], the isomorphism class of $P_{•}$, a weak U-projective resolution, in $K_{U}(R)$ only depends on the isomorphism class of *M* in the category of *R*-Mod since weak U-projective resolution is unique up to homotopy. Also notice that $P_{•}$ is a right bounded weak chain U-complex of projective modules, and hence, it's an object of $K_{U}^{-}(R-Proj)$, the homotopy category of right bounded weak chain U-complexes consist of *R*-projective modules.

Proposition 3.1 *For all R-module M, any two weak* U *-projective resolutions of M are isomorphic in* $K_{U}^{-}(R-Proj)$ *.*

Proof Suppose that $P_{•}:P_{•}(U_{•}^{P})\overset{d_{•}^{P}}{\to }M(U)$ and $Q_{•}:Q_{•}(U_{•}^{Q})\overset{d_{•}^{Q}}{\to }M(U)$ are two weak U-projective resolutions of *M*. We have

1. $U_{0}^{P}={\left(d_{0}^{P}\right)}^{-1}(U)$ and $U_{0}^{Q}={\left(d_{0}^{Q}\right)}^{-1}(U)$.
2. $U_{n}^{P}={\left(d_{n}^{P}\right)}^{-1}(U_{n-1}^{P})$ and $U_{n}^{Q}={\left(d_{n}^{Q}\right)}^{-1}(U_{n-1}^{Q})$ for every n∈N.

Now, since $P_{0}$ is projective and $d_{0}^{Q}$ is onto then there exists a morphism $f_{0}:P_{0}\to Q_{0}$ such that the diagram
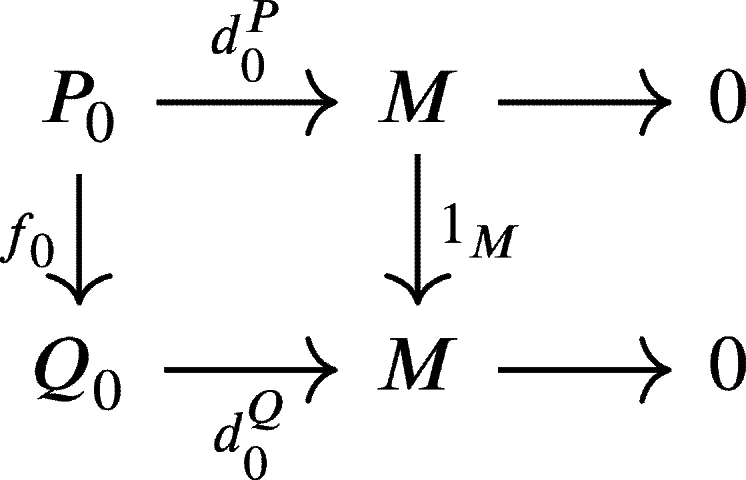
 commutes, that is $d_{0}^{Q}f_{0}=d_{0}^{P}$. Let $u_{0}\in U_{0}^{P}$. Since $d_{0}^{Q}f_{0}(u_{0})=d_{0}^{P}(u_{0})\in U$ then $f_{0}(u_{0})\in {\left(d_{0}^{Q}\right)}^{-1}(U)=U_{0}^{Q}$. Hence $f_{0}(U_{0}^{P})\subseteq U_{0}^{Q}$.

Next, since $d_{0}^{Q}f_{0}d_{1}^{P}(P_{1})=d_{0}^{P}d_{1}^{P}(P_{1})=d_{0}^{P}(\mathrm{Ker}\;d_{0}^{P}\cap U_{0}^{P})=0\subseteq U$, then f0d1P(P1)∈Kerd0Q∩U0Q=Imd1Q. And since $P_{1}$ is projective, then there exists a morphism $f_{1}:P_{1}\to Q_{1}$ such that the diagram
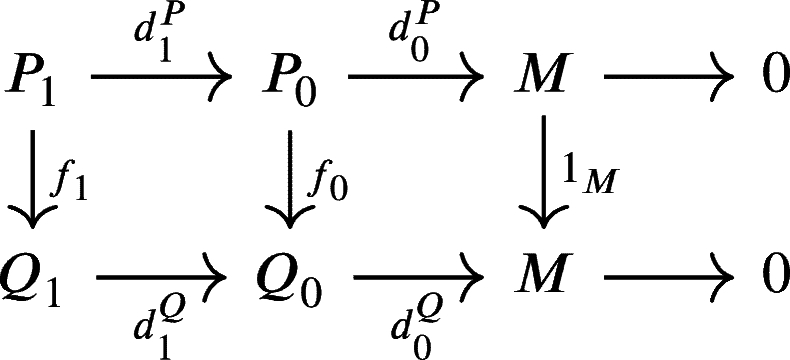
 commutes that is $d_{1}^{Q}f_{1}=f_{0}d_{1}^{P}$.

Let $u_{1}\in U_{1}^{P}={\left(d_{1}^{P}\right)}^{-1}(U_{0}^{P})$ and suppose $d_{1}^{P}(u_{1})=u_{0}\in U_{0}^{P}$ then $d_{0}^{Q}f_{0}d_{1}^{P}(u_{1})=d_{0}^{Q}f_{0}(u_{0})\in d_{0}^{Q}(U_{0}^{Q})\subseteq U$. Hence $d_{1}^{Q}f_{1}(u_{1})=f_{0}d_{1}^{P}(u_{1})\in {\left(d_{0}^{Q}\right)}^{-1}(U)=U_{0}^{Q}$ and $f_{1}(u_{1})\in {\left(d_{1}^{Q}\right)}^{-1}(U_{0}^{Q})=U_{1}^{Q}$. Therefore, $f_{1}(U_{1}^{P})\subseteq U_{1}^{Q}$.

Next, for n≥1, suppose that there exists a commutative diagram
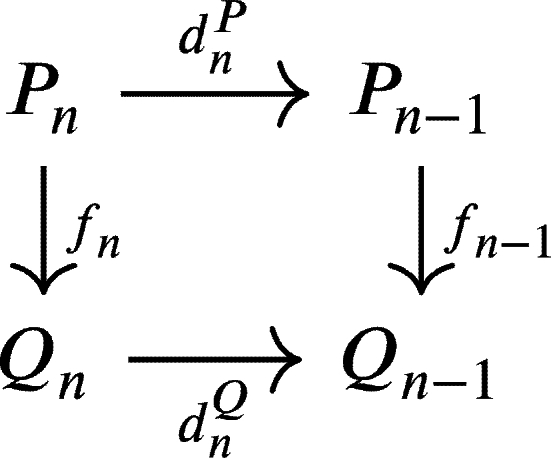
 where $f_{k}(U_{k}^{P})\subseteq U_{k}^{Q}$, for all k∈{1,⋯,n−1}. Now, let $u_{n}\in U_{n}^{P}={\left(d_{n}^{P}\right)}^{-1}(U_{n-1}^{P})$ and suppose $d_{n}^{P}(u_{n})=u_{n-1}\in U_{n-1}^{P}$, then we have

$$d_{n-1}^{Q}f_{n-1}d_{n}^{P}(u_{n})=d_{n-1}^{Q}f_{n-1}(u_{n-1})\in d_{n-1}^{Q}(U_{n-1}^{Q})\subseteq U_{n-2}^{Q}.$$

Hence

$$d_{n}^{Q}f_{n}(u_{n})=f_{n-1}d_{n}^{P}(u_{n})\in {\left(d_{n-1}^{Q}\right)}^{-1}(U_{n-2}^{Q})=U_{n-1}^{Q}$$

and $f_{n}(u_{n})\in {\left(d_{n}^{Q}\right)}^{-1}(U_{n-1}^{Q})=U_{n}^{Q}$. Therefore, we conclude that $f_{n}(U_{n}^{P})\subseteq U_{n}^{Q}$.

We have shown that for every two U-projective resolutions $P_{•}:P_{•}(U_{•}^{P})\overset{d_{•}}{\to }M(U)$ and $Q_{•}:Q_{•}(U_{•}^{Q})\overset{d_{•}}{\to }M(U)$ of *M* there exists a chain U-complex morphism $f:P_{•}\to Q_{•}$. In the same way, we can also construct a chain U-complex morphism $g:Q_{•}\to P_{•}$ and get the following commutative diagram:
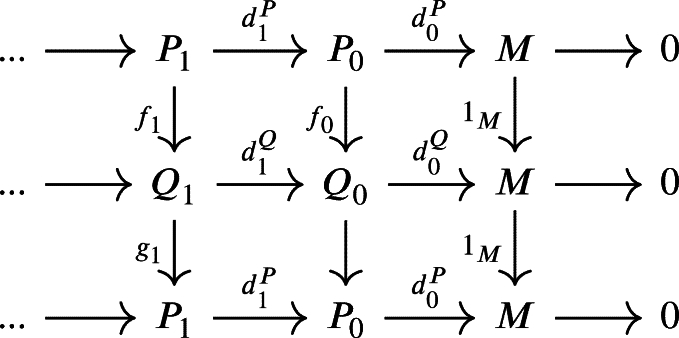
 where $f_{n}(U_{n}^{P})\subseteq U_{n}^{Q}$ and $g_{n}(U_{n}^{Q})\subseteq U_{n}^{P}$. We simplify the above diagram into
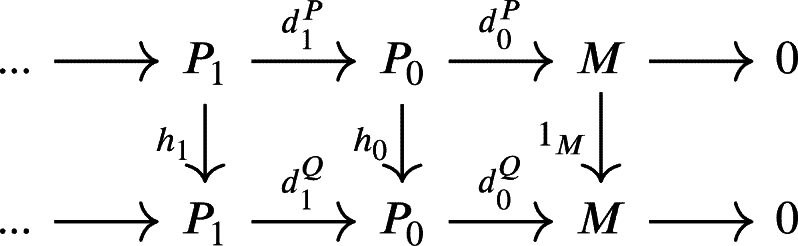
 where $h_{n}=g_{n}f_{n}$. Since $d_{0}^{P}=1_{M}d_{0}^{P}=d_{0}^{P}h_{0}$, then $d_{0}(1_{P_{0}}-h_{0})(P_{0})=0$, so that (1P0−h0)(P0)∈Kerd0P=Imd1P. Since $P_{0}$ is projective then there exists a morphism $\gamma_{1}:P_{0}\to P_{1}$ such that $1_{P_{0}}-h_{0}=d_{1}^{P}\gamma_{1}$. Define $\gamma_{0}:M\to P_{0}$ as the zero morphism, then $1_{P_{0}}-h_{0}=\gamma_{0}d_{0}^{P}+d_{1}^{P}\gamma_{1}$.

Next let $u_{0}\in U_{0}^{P}$. Since $d_{0}^{P}d_{1}^{P}\gamma_{1}(u_{0})=d_{0}^{P}(1_{P_{0}}-h_{0})(u_{0})=0$, then $d_{1}^{P}\gamma_{1}(u_{0})\in \mathrm{Ker}\;d_{0}^{P}$ and $\gamma_{1}(u_{0})\in {\left(d_{1}^{P}\right)}^{-1}(\mathrm{Ker}\;d_{0}^{P})\subseteq {\left(d_{1}^{P}\right)}^{-1}(U_{0}^{P})=U_{1}^{P}$. Hence $\gamma_{1}(U_{0}^{P})\subseteq U_{1}^{P}$.

It is clear that $d_{1}^{P}h_{1}=h_{0}d_{1}^{P}=(1_{P_{0}}-d_{1}^{P}\gamma_{1})d_{1}^{P}=d_{1}^{P}-d_{1}^{P}\gamma_{1}d_{1}^{P}$, then $d_{1}^{P}(1_{P_{1}}-\gamma_{1}d_{1}^{P}-h_{1})=0\subseteq U_{0}^{P}$ and (1P1−γ1d1P−h1)(P1)⊆Kerd1∩U1P=Imd2. Since $P_{1}$ is projective then there exists a morphism $\gamma_{2}:P_{1}\to P_{2}$ such that $d_{2}^{P}\gamma_{2}=1_{P_{1}}-\gamma_{1}d_{1}^{P}-h_{1}$ or $1_{P_{1}}-h_{1}=\gamma_{1}d_{1}^{P}+d_{2}^{P}\gamma_{2}$.

Next let $u_{1}\in U_{1}^{P}$. Since $d_{1}^{P}d_{2}^{P}\gamma_{2}(u_{1})=d_{1}^{P}(1_{P_{1}}-\gamma_{1}d_{1}^{P}-h_{1})(u_{1})=0$, then $d_{2}^{P}\gamma_{2}(u_{1})\in \mathrm{Ker}\;d_{1}^{P}$ and $\gamma_{2}(u_{1})\in {\left(d_{2}^{P}\right)}^{-1}(\mathrm{Ker}\;d_{1}^{P})\subseteq {\left(d_{2}^{P}\right)}^{-1}(U_{1}^{P})=U_{2}^{P}$. Hence $\gamma_{1}(U_{1}^{P})\subseteq U_{2}^{P}$.

Now suppose that there exists a morphism $\gamma_{n}:P_{n-1}\to P_{n}$ such that $1_{P_{n-1}}-h_{n-1}=\gamma_{n-1}d_{n-1}^{P}+d_{n}^{P}\gamma_{n}$ and $\gamma_{n}(U_{n-1}^{P})\subseteq U_{n}^{P}$. Then $d_{n}^{P}h_{n}=h_{n-1}d_{n}^{P}=(1_{P_{n-1}}-d_{n}^{P}\gamma_{n})d_{n}^{P}=d_{n}^{P}-d_{n}^{P}\gamma_{1}d_{n}^{P}$, then $d_{n}^{P}(1_{P_{n}}-\gamma_{n}d_{n}^{P}-h_{n})=0\subseteq U_{n-1}^{P}$ and (1Pn−γndnP−hn)(Pn)⊆Kerdn∩UnP=Imdn+1. Since $P_{n}$ is projective then there exists a morphism $\gamma_{n+1}:P_{n}\to P_{n+1}$ such that $d_{n+1}^{P}\gamma_{n+1}=1_{P_{n}}-\gamma_{n}d_{n}^{P}-h_{n}$ or $1_{P_{n}}-h_{n}=\gamma_{n}d_{n}^{P}+d_{n+1}^{P}\gamma_{n+1}$.

Next let $u_{n}\in U_{n}^{P}$. Since $d_{n}^{P}d_{n+1}^{P}\gamma_{n+1}(u_{n})=d_{n}^{P}(1_{P_{n}}-\gamma_{n}d_{n}^{P}-h_{n})(u_{n})=0$, then $d_{n+1}^{P}\gamma_{n+1}(u_{n})\in \mathrm{Ker}\;d_{n}^{P}$ and $\gamma_{n+1}(u_{n})\in {\left(d_{n+1}^{P}\right)}^{-1}(\mathrm{Ker}\;d_{n}^{P})\subseteq {\left(d_{n+1}^{P}\right)}^{-1}(U_{n}^{P})=U_{n+1}^{P}$. Hence $\gamma_{n}(U_{n}^{P})\subseteq U_{n+1}^{P}$.

We conclude that $\mathrm{gf}∼1_{P_{•}}$ and similarly we can show that $\mathrm{fg}∼1_{Q_{•}}$ Therefore, the U-projective resolution is unique up to homotopy. □

Proposition 3.2 *By defining a functor that associates a weak* U *-projective resolution to each R-module, the category R-Mod is a full subcategory of* $K_{U}^{-}(R-Proj)$ *.*

## 4 Derived category of weak chain U-complexes

We now wish to obtain the derived category $D_{U}(R)$ by formally inverting a class of morphisms in $K_{U}(R)$. Throughout this section, the objects in $D_{U}^{-}(R)$, $D_{U}^{+}(R)$, and $D_{U}^{b}(R)$ are right bounded weak chain U-complexes of *R*-modules, left bounded weak chain U-complexes of *R*-modules, and left and right bounded weak chain U-complexes of *R*-modules, respectively. We will then describe the morphisms in those categories.

Definition 4.1 Let *X* and *Y* be weak chain U-complexes and let f:X→Y be a morphism of weak chain U-complexes. We call *f* a quasi-isomorphism if H(f):H(X)→H(Y) is an isomorphism.

Theorem 4.2 *Let* $0\to X\overset{f}{\to }Y\overset{g}{\to }Z\to 0$ *be an exact sequence of weak chain* U *-complexes* X,Y *and Z, where for all* n∈Z

1. $0\to X_{n}\overset{f_{n}}{⟶}Y_{n}\overset{g_{n}}{⟶}Z_{n}\to 0$ *is split exact.*
2. $0\to U_{n}^{X}\overset{f_{n}^{\prime }}{⟶}U_{n}^{Y}\overset{g_{n}^{\prime }}{⟶}U_{n}^{Z}\to 0$ *, is split exact, where* $f_{n}^{\prime }=f_{n}{|}_{U_{n}^{X}}$ *and* $g_{n}^{\prime }=g_{n}{|}_{U_{n}^{Y}}$ *.*

*Then there exists a long exact sequence*

$$\ldots \to H_{n}(X)\overset{\bar{f_{n}}}{\to }H_{n}(Y)\overset{\bar{g_{n}}}{\to }H_{n}(Z)\overset{\delta_{n}}{\to }H_{n-1}(X)\overset{\bar{f_{n-1}}}{\to }H_{n-1}(Y)\overset{\bar{g_{n-1}}}{\to }H_{n-1}(Z)\to \ldots ,$$

*where* $\bar{f_{n}}=H_{n}(f)$ *and* $\bar{g_{n}}=H_{n}(g)$ *for all* n∈Z *.*

Note that we can expand the first exact sequence as follows.
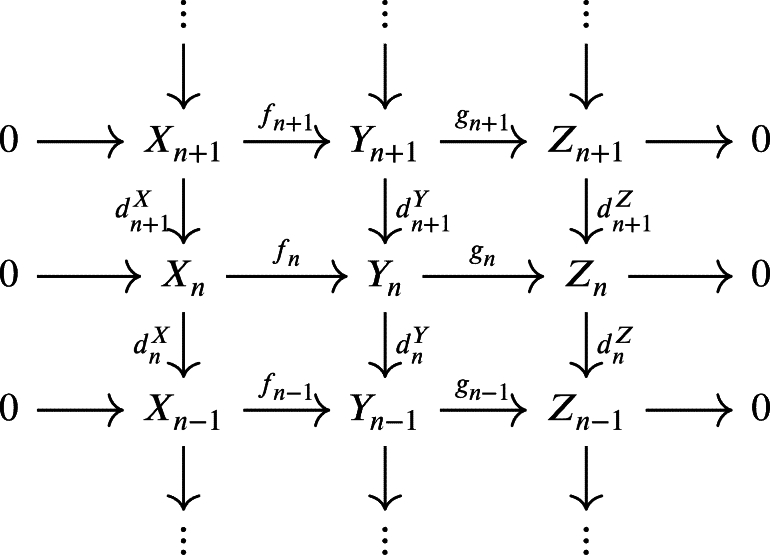


The proof of Theorem 4.2 will use the following Lemma 4.3.

Lemma 4.3 *Assume the hypothesis of*Theorem 4.2*, for all*n∈Z*there exists an exact sequence*

Hn(X)=dn−1(Un−1X)UnX+Imdn+1X⟶fn‾Hn(Y)=dn−1(Un−1Y)UnY+Imdn+1Y⟶gn‾Hn(Z)=dn−1(Un−1Z)UnZ+Imdn+1Z.

Proof We will show that Imfn‾=Kergn‾. Let yn+UnY+Imdn+1Y∈Imfn‾. Then by Lemma 2.13 and Theorem 2.14

yn+UnY+Imdn+1Y=fn‾(xn+UnX+Imdn+1X)=fn(xn)+UnY+Imdn+1Y.,

for some $x_{n}\in d_{n}^{-1}(U_{n-1}^{X})$ So, $y_{n}=f_{n}(x_{n})+u_{n}^{Y}+d_{n+1}^{Y}(y_{n+1})$ for some $u_{n}^{Y}\in U_{n}^{Y}$ and $y_{n+1}\in Y_{n+1}$. Now also by Lemma 2.13 and Theorem 2.14 we have

gn‾(yn+UnY+Imdn+1Y)=gn(yn)+UnZ+Imdn+1Z=gn(fn(xn)+unY+dn+1Y(yn+1))+UnZ+Imdn+1Z=gndn+1Y(yn+1)+UnZ+Imdn+1Z=dn+1Zgn+1(yn+1)+UnZ+Imdn+1Z=UnZ+Imdn+1Z.

Hence, we have Imfn‾⊆Kergn‾.

Next, we need to show the reverse inclusion. Take yn+UnY+Imdn+1Y∈Kergn‾, then gn‾(yn+UnY+Imdn+1Y)=0‾. In other word, gn(yn)∈UnZ+Imdn+1Z. Thus, we have

$$g_{n}(y_{n})=u_{n}^{Z}+d_{n+1}^{Z}(z_{n+1})\;\text{ for some }u_{n}^{Z}\in U_{n}^{Z}\text{ and }z_{n+1}\in Z_{n+1}=u_{n}^{Z}+d_{n+1}^{Z}(g_{n+1}(y_{n+1}))\;\text{ for some }y_{n+1}\in Y_{n+1}=u_{n}^{Z}+g_{n}d_{n+1}^{Y}(y_{n+1})=g_{n}(u_{n}^{Y})+g_{n}d_{n+1}^{Y}(y_{n+1})\;\text{ for some }u_{n}^{Y}\in U_{n}^{Y}$$

Hence, yn−unY−dn+1Y(yn+1)∈Kergn=Imfn. In other words, $y_{n}-u_{n}^{Y}-d_{n+1}^{Y}(y_{n+1})=f_{n}(x_{n})$, for some $x_{n}\in X_{n}$. Therefore, we have

yn+UnY+Imdn+1Y=fn‾(xn+UnX+Imdn+1X),

and thus Kergn‾⊆Imfn‾. Joining this result with the previous one, we conclude that Imfn‾=Kergn‾, thus showing the exactness. □

Proof of Theorem 4.2 First, we need to construct the connecting morphism $\delta_{n}$ to prove the theorem. After that, we can then show the exactness of the sequence. We only need to show the exactness at $\bar{f_{n}}$, $\bar{g_{n}}$, $\delta_{n}$, $\bar{f_{n-1}}$, and $\bar{g_{n-1}}$. The rest of the sequence follows from the generality of the argument.

1. Construction of $\delta_{n}$.
  Let zn+UnZ+Imdn+1Z∈Hn(Z). Here, $z_{n}\in d_{n}^{-1}(U_{n-1}^{Z})$. Since $g_{n}$ is surjective, there exists $y_{n}\in Y_{n}$ such that $g_{n}(y_{n})=z_{n}$. Now, notice that
  $$\begin{array}{ccc} g_{n-1}d_{n}^{Y}(y_{n})\; & =\; & d_{n}^{Z}g_{n}(y_{n})\\ \; & =\; & d_{n}^{Z}(z_{n})\in U_{n-1}^{Z}\\ \; & =\; & g_{n-1}(u_{n-1}^{Y}) \end{array}$$
  for some $u_{n-1}^{Y}\in U_{n-1}^{Y}$ since $g_{n-1}{|}_{U_{n-1}^{Y}}$ is surjective.
  So, we have $g_{n-1}(d_{n}^{Y}(y_{n})-u_{n-1}^{Y})=0$ and dnY(yn)−un−1Y∈Imfn−1.
  Since $f_{n-1}$ is injective, then there exists a unique $x_{n-1}\in X_{n-1}$ such that
  (3) $$f_{n-1}(x_{n-1})=d_{n}^{Y}(y_{n})-u_{n-1}^{Y}.$$
  Now we will show that $d_{n-1}^{X}(x_{n-1})\in U_{n-2}^{X}$. We have
  $$\begin{array}{ccc} f_{n-2}d_{n-1}^{X}(x_{n-1})\; & =\; & d_{n-1}^{Y}f_{n-1}(x_{n-1})\\ \; & =\; & d_{n-1}^{Y}(d_{n}^{Y}(y_{n})-u_{n-1}^{Y})\in U_{n-2}^{Y}. \end{array}$$
  Since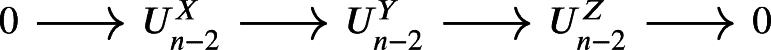 is a split exact sequence, then $f_{n-2}$ has a left inverse. Therefore, we have $d_{n-1}^{X}(x_{n-1})\in U_{n-2}^{X}$. Hence, we can define
  δn(zn+UnZ+Imdn+1Z)=xn−1+Un−1X+ImdnX.
  Next, we will show that $\delta_{n}$ is well-defined. Let $y_{n}^{\prime }\in Y_{n}$ such that $g_{n}(y_{n}^{\prime })$ is also equal to $z_{n}$. Then, there exists $x_{n-1}^{\prime }\in X_{n-1}$ such that
  $$f_{n-1}(x_{n-1}^{\prime })=d_{n}^{Y}(y_{n}^{\prime })-u_{n-1}^{\prime \;Y},$$
  for some $u_{n-1}^{\prime \;Y}\in U_{n-1}^{Y}$. We will show that xn−1−xn−1′∈ImdnX+Un−1X. Since $0=g_{n}(y_{n}-y_{n}^{\prime })$, then $y_{n}-y_{n}^{\prime }=f_{n}(\bar{x_{n}})$ for some $\bar{x_{n}}\in X_{n}$. Let $L_{n-1}^{Y}$ be the complement of $f_{n-1}(U_{n-1}^{X})$ in $U_{n-1}^{Y}$. We have
  $$\begin{array}{ccc} f_{n-1}(x_{n-1}-x_{n-1}^{\prime })\; & =\; & d_{n}^{Y}(y_{n})-u_{n-1}^{Y}-d_{n}^{Y}(y_{n}^{\prime })+u_{n-1}^{\prime \;Y}\\ \; & =\; & d_{n}^{Y}(y_{n}-y_{n}^{\prime })-(u_{n-1}^{Y}-u_{n-1}^{\prime \;Y})\\ \; & =\; & d_{n}^{Y}(f_{n}(\bar{x_{n}}))-(u_{n-1}^{Y}-u_{n-1}^{\prime \;Y})\\ \; & =\; & f_{n-1}d_{n}^{X}(\bar{x_{n}})+f_{n-1}({\bar{u_{n-1}}}^{X})+l_{n-1}^{Y} \end{array}$$
  for some ${\bar{u_{n-1}}}^{X}\in U_{n-1}^{X},l_{n-1}^{Y}\in L_{n-1}^{Y}$.
  Since $0\to U_{n-1}^{X}\to U_{n-1}^{Y}\to U_{n-1}^{Z}\to 0$ is split exact, then $f_{n-1}$ has a left inverse, call it $\gamma_{n-1}$. We then have
  $$\begin{array}{ccc} \gamma_{n-1}f_{n-1}(x_{n-1}-x_{n-1}^{\prime })\; & =\; & \gamma_{n-1}f_{n-1}d_{n}^{X}(\bar{x_{n}})+\gamma_{n-1}f_{n-1}({\bar{u_{n-1}}}^{X})+\gamma_{n-1}(l_{n-1}^{Y})\\ x_{n-1}-x_{n-1}^{\prime }\; & =\; & d_{n}^{X}(\bar{x_{n}})+{\bar{u_{n-1}}}^{X}+\gamma_{n-1}(l_{n-1}^{Y}) \end{array}$$
  Therefore, xn−1−xn−1′∈ImdnX+Un−1X. We then conclude that $\delta_{n}$ is well-defined.
2. We will show that Imgn‾=Kerδn. Let zn+UnZ+Imdn+1Z∈Imgn‾, with $z_{n}\in {\left(d_{n}^{Z}\right)}^{-1}(U_{n-1}^{Z})$. Then
  (4) zn+UnZ+Imdn+1Z=gn‾(yn+UnY+Imdn+1Y)
  for some $y_{n}\in {\left(d_{n}^{Y}\right)}^{-1}(U_{n-1}^{Y})$. We have
  δn(zn+UnZ+Imdn+1Z)=δn(gn‾(yn+UnY+Imdn+1Y))=δn(gn(yn)+UnZ+Imdn+1Z)=xn−1+Un−1X+ImdnX,
  for some $x_{n-1}$ such that $f_{n-1}(x_{n-1})=d_{n}^{Y}(y_{n})-u_{n-1}^{Y}$, where $u_{n-1}^{Y}$ is any element of $U_{n-1}^{Y}$ such that $g_{n-1}d_{n}^{Y}=g_{n-1}(u_{n-1}^{Y})$.
  We will show that xn−1∈Un−1X+ImdnX. By (4), zn+UnZ+Imdn+1Z=gn(yn)+UnZ+Imdn+1Z, then zn−gn(yn)∈UnZ+Imdn+1Z. Hence, we have
  $$\begin{array}{ccc} z_{n}\; & =\; & g_{n}(y_{n})+u_{n}^{Z}+d_{n+1}^{Z}(z_{n+1})\;\text{for some}\;u_{n}^{Z}\in U_{n}^{Z},z_{n+1}\in Z_{n+1}\\ \; & =\; & g_{n}(y_{n}+u_{n}^{Y}+y_{n}^{\prime \prime })\;\text{for some}\;u_{n}^{Y}\in U_{n}^{Y},y_{n}^{\prime \prime }\in Y_{n}\;\text{where}\;g_{n}(y_{n}^{\prime \prime })=d_{n+1}^{Z}(z_{n+1}) \end{array}$$
  But since $g_{n}$ is surjective, we have $z_{n}=g_{n}(y_{n}^{\prime })$ for some $y_{n}^{\prime }\in Y_{n}$. Hence
  $$\begin{array}{cc} y_{n}^{\prime }-y_{n}-u_{n}^{Y}-y_{n}^{\prime \prime }\; & \in \mathrm{Ker}\;g_{n}\\ y_{n}^{\prime }-y_{n}-u_{n}^{Y}-y_{n}^{\prime \prime }\; & =f_{n}(\bar{x_{n}})\;\text{for some}\;\bar{x_{n}}\in X_{n}\\ \text{So,}\;d_{n}^{Y}(f_{n}(\bar{x_{n}}))\; & =d_{n}^{Y}(y_{n}^{\prime }-y_{n}-u_{n}^{Y}-y_{n}^{\prime \prime })\\ f_{n-1}(x_{n-1})\; & =d_{n}^{Y}(y_{n})-u_{n-1}^{Y}\\ \; & =d_{n}^{Y}(-f_{n}(\bar{x_{n}}))+d_{n}^{Y}(y_{n}^{\prime })-d_{n}^{Y}(u_{n}^{Y})-d_{n}^{Y}(y_{n}^{\prime \prime })-u_{n-1}^{Y} \end{array}$$
  Let $L_{j}^{Y}$ be the complement of $f_{j}(X_{j})$ in $Y_{j}$ and $L_{j}^{U}$ be the complement of $f_{j}(U_{j}^{X})$ in $U_{j}^{Y}$, for all j∈Z. We have
  • $y_{n}^{\prime }-y_{n}^{\prime \prime }=f_{n}(x_{n}^{\prime \prime })+l_{n}^{Y}$ for some $x_{n}^{\prime \prime }\in X_{n},l_{n}^{Y}\in L_{n}^{Y}$,
  • $u_{j}^{Y}=f_{j}(u_{j}^{X})+l_{j}^{U}$ for some $u_{j}^{X}\in U_{j}^{X},l_{j}^{U}\in L_{j}^{U}$.
  Therefore,
  $$\begin{array}{ccc} f_{n-1}(x_{n-1})\; & =\; & d_{n}^{Y}(-f_{n}(\bar{x_{n}}))+d_{n}^{Y}(f_{n}(x_{n}^{\prime \prime })+l_{n}^{Y})-d_{n}^{Y}(f_{n}(u_{n}^{X})+l_{n}^{U})-f_{n-1}(u_{n-1}^{X})-l_{n-1}^{U}\\ \; & =\; & f_{n-1}(d_{n}^{X}(-\bar{x_{n}}+x_{n}^{\prime \prime }-u_{n}^{X})-u_{n-1}^{X})+d_{n}^{Y}(l_{n}^{Y}-l_{n}^{U})-l_{n-1}^{U} \end{array}$$
  Since $f_{n-1}$ has a left inverse, say $f_{n-1}^{\prime }$, we have
  $$f_{n-1}^{\prime }f_{n-1}(x_{n-1})=f_{n-1}^{\prime }f_{n-1}(d_{n}^{X}(-\bar{x_{n}}+x_{n}^{\prime \prime }-u_{n}^{X})-u_{n-1}^{X})+f_{n-1}^{\prime }(d_{n}^{Y}(l_{n}^{Y}-l_{n}^{U})-l_{n-1}^{U}).$$
  Thus, xn−1∈ImdnX+Un−1X. We then conclude that Imgn‾⊆Kerδn.
  Conversely, let zn+UnZ+Imdn+1Z∈Kerδn. We have
  δn(zn+UnZ+Imdn+1Z)=xn−1+Un−1X+ImdnX=Un−1X+ImdnX
  So xn−1∈Un−1X+ImdnX. In other words, $x_{n-1}=u_{n-1}^{X}+d_{n}^{X}(x_{n})$ for some $u_{n-1}^{X}\in U_{n-1}^{X}$ and $x_{n}\in X_{n}$.
  Now, using the construction of $\delta_{n}$, we have $z_{n}=g_{n}(y_{n})$ and $f_{n-1}(x_{n-1})=d_{n}^{Y}(y_{n})-u_{n-1}^{Y}$. Hence,
  $$\begin{array}{ccc} d_{n}^{Y}(y_{n})\; & =\; & f_{n-1}(u_{n-1}^{X}+d_{n}^{X}(x_{n}))+u_{n-1}^{Y}\\ \; & =\; & f_{n-1}(u_{n-1}^{X})+f_{n-1}d_{n}^{X}(x_{n})+u_{n-1}^{Y} \end{array}$$
  So, $d_{n}^{Y}(y_{n})-d_{n}^{Y}(f_{n}(x_{n}))\in U_{n-1}^{Y}$ and $y_{n}-f_{n}(x_{n})\in d_{n}^{-1Y}(U_{n-1}^{Y})$. Therefore, there exists $y_{n}-f_{n}(x_{n})\in Y_{n}$ such that
  gn‾(yn−fn(xn)+UnY+Imdn+1Y)=gn(yn)+UnZ+Imdn+1Z=zn+UnZ+Imdn+1Z.
  We conclude that Kerδn⊆Imgn‾. Hence, we have Imgn‾=Kerδn.
3. We will show that Imδn=Kerfn−1‾.
  Let xn−1+Un−1X+ImdnX∈Imδn. Then there exists zn+UnZ+Imdn+1Z such that
  δn(zn+UnZ+Imdn+1Z)=xn−1+Un−1X+ImdnX.
  We will show that
  fn−1‾(xn−1+Un−1X+ImdnX)=Un−1Y+ImdnY.
  By Lemma 4.3, we have
  fn−1‾(xn−1+Un−1X+ImdnX)=fn−1(xn−1)+Un−1Y+ImdnY.
  By (3) in the construction of $\delta_{n}$, fn−1(xn−1)=dnY(yn)−un−1Y∈ImdnY+Un−1Y. Hence Imδn⊆Kerfn−1‾.
  Conversely, let xn−1+Un−1X+ImdnX∈Kerfn−1‾. Then
  $$f_{n-1}(x_{n-1})=u_{n-1}^{Y}+d_{n}^{Y}(y_{n})$$
  for some $u_{n-1}^{Y}\in U_{n-1}^{Y},y_{n}\in Y_{n}$. So,
  un−1Y+dnY(yn)∈Imfn−1=Kergn−1,
  and then
  $$\begin{array}{ccc} g_{n-1}(u_{n-1}^{Y})\; & =\; & -g_{n-1}d_{n}^{Y}(y_{n})\\ \; & =\; & -d_{n}^{Z}g_{n}(y_{n}). \end{array}$$
  Now choose $z_{n}=g_{n}(y_{n})$. First show that $z_{n}\in d_{n}^{-1}(U_{n-1}^{Z})$. We have
  $$\begin{array}{ccc} d_{n}(z_{n})\; & =\; & d_{n}^{Z}g_{n}(y_{n})\\ \; & =\; & g_{n-1}d_{n}^{Y}(y_{n})\\ \; & =\; & g_{n-1}(u_{n-1}^{Y})\in U_{n-1}^{Z}. \end{array}$$
  Now we will show that δn(zn+UnZ+Imdn+1Z)=xn−1+Un−1X+ImdnX.
  Let $y_{n}^{\prime }\in Y_{n}$ such that $g_{n}(y_{n}^{\prime })=z_{n}$. By the construction of $\delta_{n}$, there exists $x_{n-1}^{\prime }\in X_{n-1}$ such that $f_{n-1}(x_{n-1}^{\prime })=d_{n}^{Y}(y_{n}^{\prime })-u_{n-1}^{\prime \;Y}$ for some $u_{n-1}^{\prime \;Y}\in U_{n-1}^{Y}$.
  We will show that xn−1−xn−1′∈Un−1X+ImdnX. We have
  (5) $$f_{n-1}(x_{n-1}-x_{n-1}^{\prime })=f_{n-1}(x_{n-1})-f_{n-1}(x_{n-1}^{\prime })=u_{n-1}^{Y}+d_{n}^{Y}(y_{n})-(d_{n}^{Y}(y_{n}^{\prime })-u_{n-1}^{\prime \;Y}).$$
  Hence un−1Y+dnY(yn)−(dnY(yn′)−un−1′Y)∈Imfn−1=Kergn−1, and we have
  $$\begin{array}{ccc} 0\; & =\; & g_{n-1}(u_{n-1}^{Y}+d_{n}^{Y}(y_{n})-(d_{n}^{Y}(y_{n}^{\prime })-u_{n-1}^{\prime \;Y})\\ \; & =\; & g_{n-1}(u_{n-1}^{Y}+u_{n-1}^{\prime \;Y})+g_{n-1}d_{n}^{Y}(y_{n}-y_{n}^{\prime })\\ \; & =\; & g_{n-1}(u_{n-1}^{Y}+u_{n-1}^{\prime \;Y})+d_{n}^{Z}g_{n}(y_{n}-y_{n}^{\prime })\\ \; & =\; & g_{n-1}(u_{n-1}^{Y}+u_{n-1}^{\prime \;Y}). \end{array}$$
  Hence un−1Y+un−1′Y∈Kergn−1=Imfn−1, and there exists $u_{n-1}^{X}$ such that
  (6) $$f_{n-1}(u_{n-1}^{X})=u_{n-1}^{Y}+u_{n-1}^{\prime \;Y}.$$
  Notice that since $g_{n}(y_{n}-y_{n}^{\prime })=0$, then yn−yn′∈Kergn=Imfn. Hence $y_{n}-y_{n}^{\prime }=f_{n}(x_{n}-x_{n}^{\prime })$ for some $x_{n},x_{n}^{\prime }\in X_{n}$. We get
  (7) $$d_{n}(y_{n}-y_{n}^{\prime })=d_{n}f_{n}(x_{n}-x_{n}^{\prime })=f_{n-1}(d_{n}(x_{n}-x_{n}^{\prime })).$$
  Combining (5), (6), (7), we obtain
  $$\begin{array}{ccc} f_{n-1}(x_{n-1}-x_{n-1}^{\prime })\; & =\; & u_{n-1}^{Y}+d_{n}^{Y}(y_{n})-(d_{n}^{Y}(y_{n}^{\prime })-u_{n-1}^{\prime \;Y})\\ \; & =\; & f_{n-1}(u_{n-1}^{X})+f_{n-1}(d_{n}(x_{n}-x_{n}^{\prime })). \end{array}$$
  Since $f_{n-1}$ is injective, then
  xn−1−xn−1′=un−1X+dn(xn−xn′)∈Un−1X+ImdnX.
  We then have δn(zn+UnZ+Imdn+1Z)=xn−1′+Un−1X+ImdnX=xn−1+Un−1X+ImdnX and then Kerfn−1‾⊆Imδn. Therefore Imδn=Kerfn−1‾. This concludes our proof. □

Corollary 4.4 *Let* f:X→Y *be a homomorphism of weak chain* U *-complexes with* Z=M(f) *. Then*

1. *there is a long exact sequence*
  $$\cdots \to H_{i+1}(Z)\to H_{n}(X)\to H_{n}(Y)\to H_{n}(Z)\to H_{i-1}(X)\to \cdots$$
  *for all* i∈Z *.*
2. M(f) *is acyclic (i.e., having zero homology on all degrees) if and only if f is a quasi-isomorphism.*

### 4.1 Construction of derived category of weak chain U-complexes

Given weak chain U-complexes *X* and *Y*, let us consider triples
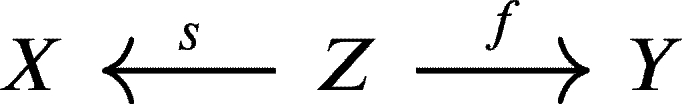
 where *Z* is any weak chain U-complex and f,s are homomorphisms of weak chain U-complexes.

Two triples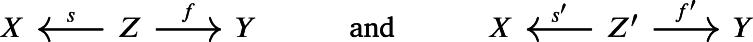 are equivalent if there exists a triple
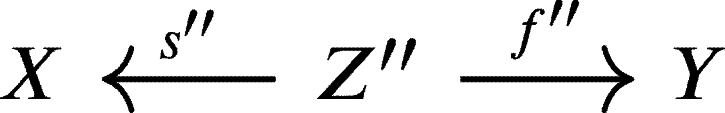
 such that the following diagram commutes:
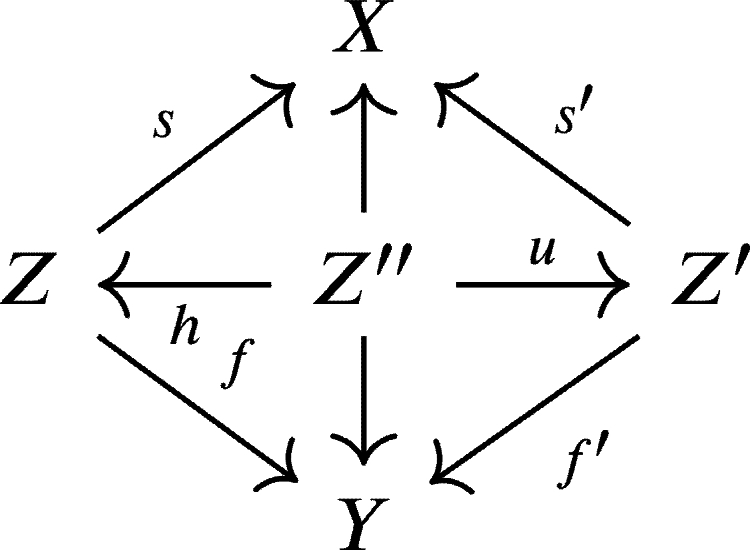


For two triples 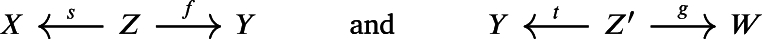 we will find an object $Z^{\prime \prime }$ together with morphisms $Z\leftarrow Z^{\prime \prime }\to Z$ which yields the following “roof” diagram
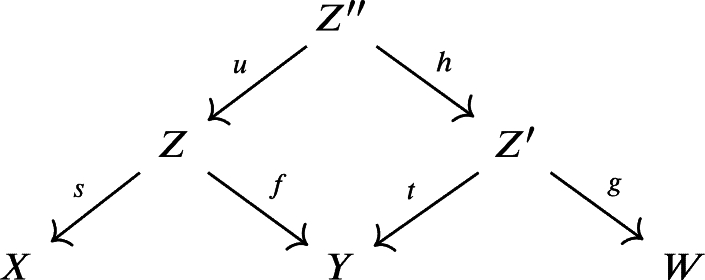
 and then we define the triple
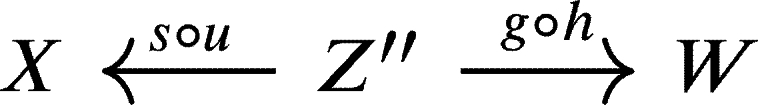
 as the composition of
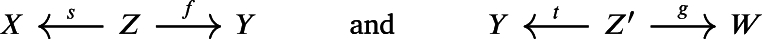

Lemma 4.5 *Let* Z,Y *, and* $Z^{\prime }$ *be objects in* $K_{U}(R)$ *together with morphism* Z→Y *and a quasi-isomorphism* $Z^{\prime }\to Y$ *. There exists an object* $Z^{\prime \prime }$ *in* $K_{U}(R)$ *, a quasi-isomorphism* $Z^{\prime \prime }\to Z$ *, and a morphism* $Z^{\prime \prime }\to Z^{\prime }$ *such that the square*

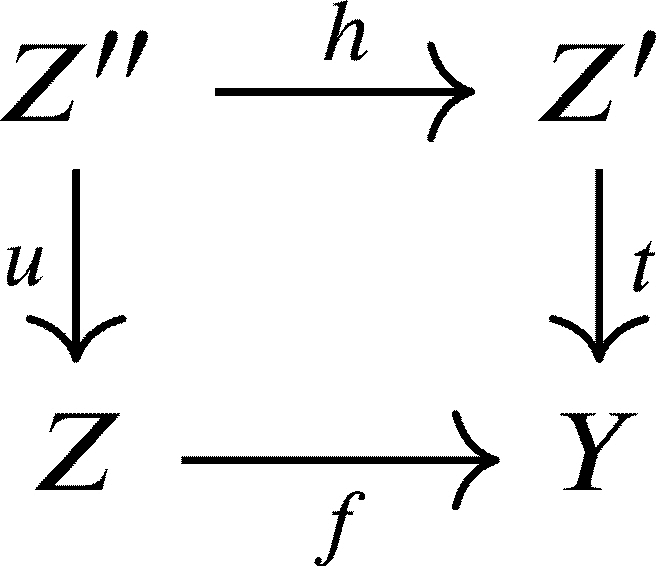

*commutes.*

Proof Observe the following diagram, where M(t) is the mapping cone of *t* as defined in Definition 2.19:
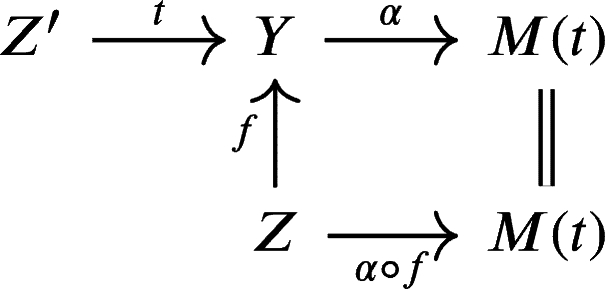

We can form cones to complete the previous diagram into this diagram
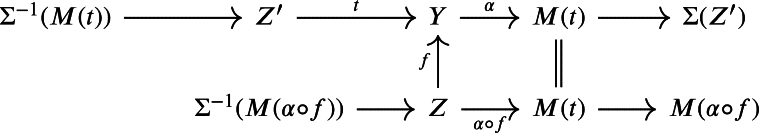
 of distinguished triangles such that the middle square commutes. The second line

$$\Sigma^{-1}(M(\alpha \circ f))\to Z\overset{\alpha \circ f}{\to }M(t)\to M(\alpha \circ f)$$

is a distinguished triangle by Theorem 2.26, TR2, and TR3 of Definition 2.25.

Since $K_{U}(R)$ is triangulated, then by TR4 of Definition 2.25, there exists a mapping *h* making the diagram
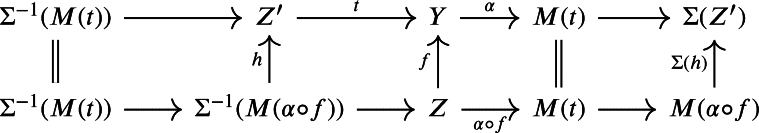
 commutes. Define

$$Z^{\prime \prime }=\Sigma^{-1}M(\alpha \circ f),\;\;u:Z^{\prime \prime }\to Z,\;\;\text{and}\;\;h:Z^{\prime \prime }\to Z^{\prime }$$

as given in the above diagram. Since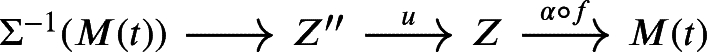 and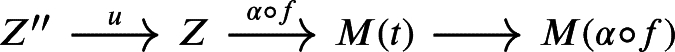 are distinguished triangle, then by Definition 2.24 the cone of *u* is isomorphic to M(t) in $K_{U}(R)$. If *t* is a quasi-isomorphism, then by Corollary 4.4, M(t) is acyclic, and we also conclude that *u* is a quasi-isomorphism. □

Remark 4.6 We cannot simply use the pullback because we need $u:Z^{\prime \prime }\to Z$ to be a quasi-isomorphism.

Definition 4.7 The derived category of weak chain U-complexes, denoted by $D_{U}(R)$, has weak U-complexes chain of *R*-modules as its objects. For any two objects *X* and *Y* in $D_{U}(R)$, ${\mathrm{Hom}}_{D_{U}(R)}(X,Y)$ is defined as the set of equivalence classes of triples $X\overset{s}{\leftarrow }Z\overset{f}{\to }Y$ where *Z* is an object of $D_{U}(R)$, *s* is a quasi-isomorphism and *f* is a morphism of weak chain U-complexes.

Remark 4.8 The classical derived category is only defined for an exact category with specific condition [9]. However, inverting the morphism can be applied for any category, not just triangulated categories. In particular, for triangulated categories, we can invert the morphisms that form a multiplicative system. We use the word derived because the construction is similar to the classical derived category construction. The construction of classical derived category does require the category to be an abelian category. The category we construct does not require that condition.

For x,y∈{,+,−,b} we denote by $D_{U}^{x,y}(R)$ the full subcategory of $D_{U}(R)$ formed by objects in $K_{U}^{x,y}(R)$.

Proposition 4.9 *For* x,y∈{∅,+,−,b} *the category* $D_{U}^{x,y}(R)$ *together with the suspension functor induced by the homotopy category* $K_{U}^{x,y}(R)$ *is a triangulated category. The distinguished triangles are all triangles isomorphic to a triangle of the form*

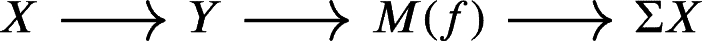

Corollary 4.10 *We obtain a functor of triangulated categories N, which is the natural functor*$N:K_{U}^{x,y}(R)\to D_{U}^{x,y}(R)$*given by sending a weak chain*U*-complex X to itself (in*$D_{U}^{x,y}(R)$*) and a morphism*f:X→Y*to the triple*$\left(id_{X},X,f\right)$*. The subcategory formed by the objects of*$K_{U}^{x,y}(R)$*that is mapped to weak chain*U*-complexes isomorphic to 0 in*$D_{U}^{x,y}(R)$*is the full subcategory of*$K_{U}^{x,y}(R)$*formed by acyclic weak chain*U*-complexes. Given a triangulated category*C*and a functor*$F:K_{U}^{x,y}(R)\to C$*that satisfies:*

*a*. *maps distinguished triangles to distinguished triangles,*
*b*. *commutes with the suspension functors of the two triangulated categories, and*
*c*. F(s)*is invertible in*C*, for every quasi-isomorphism s in*$K_{U}^{x,y}(R)$*.*

*then there exists a functor*$G:D_{U}^{x,y}(R)\to C$*, such that*F=G∘N*.*

The proof of Proposition 4.9 and Corollary 4.10 are similar to the proof of [10, Proposition 3.5.40 and Corollary 3.5.41].

### 4.2 Projective resolutions

Now we will identify an object in the derived category of weak chain U-complex as an object in the right bounded homotopy category of weak chain U-complexes of projective modules.

Definition 4.11 Let *X* be an object in $D_{U}^{-}(R)$. A projective resolution of *X* is an object $P_{X}$ in $K_{U}^{-}($R−Proj) such that $P_{X}≅X$ in $D_{U}^{-}(R)$.

Proposition 4.12 *Let X be an object in* $D_{U}^{-}(R)$ *. The projective resolution of X exists.*

Proof Without loss of generality, we may assume that *X* is isomorphic to some weak chain U-complex whose negative degree components are all zero. Now, if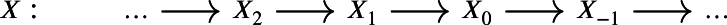 is a weak chain U-complex such that $H_{i}(X)=0$ for i<0, then
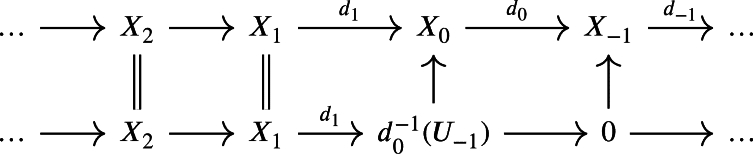
 there is a quasi-isomorphism between these two complexes. Hence, assume that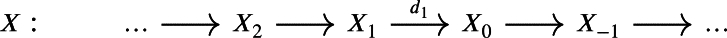 is a complex such that $d_{1}$ is not surjective. Let $P_{0}$ be a projective module along with a projection $\mu_{0}:P_{0}\to X_{0}$. We then form the pullback with $d_{1}$ which yields the following diagram:
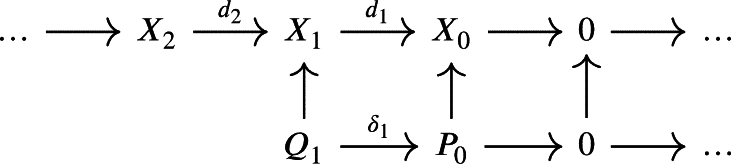

In general, $Q_{1}$ will not be projective. We can solve this by taking a projective module $P_{1}$ along with a projection $\mu_{1}:P_{1}\to Q_{1}$ and form the pullback with $d_{2}$
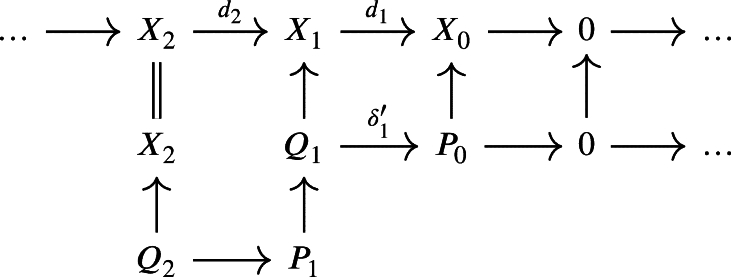
 which gives a weak chain U-complex in the second line
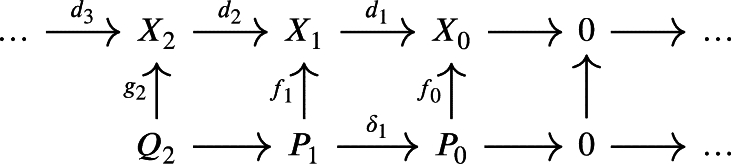
 for a certain mapping $\delta_{1}$ obtained by composing $\delta_{1}^{\prime }$ with $\mu_{1}$ and by defining $U_{0}^{P}=f_{0}^{-1}(U_{0}^{X})$ and $U_{1}^{P}=f_{1}^{-1}(U_{1}^{X})\cap \delta_{1}^{-1}(U_{0}^{P})$.

We continue with a projective module $P_{2}$ along with a projection $\mu_{2}:P_{2}\to Q_{2}$ and take the pullback along $d_{3}$ to obtain
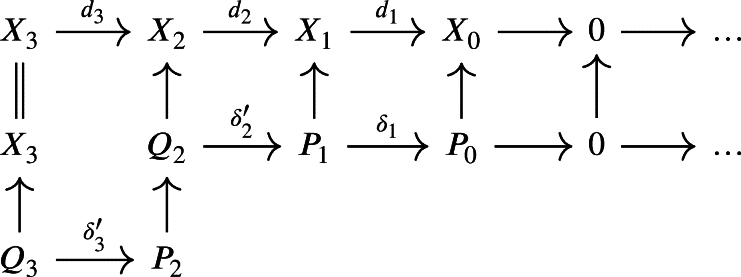
 which gives then, composing $\delta_{2}^{\prime }$ with $\mu_{2}$ to get $\delta_{2}$
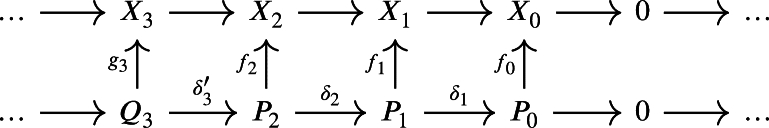
 and we define $U_{2}^{P}=f_{2}^{-1}(U_{2}^{X})\cap \delta_{2}^{-1}(U_{1}^{P})$.

Inductively, we obtain a weak chain U-complex $\left(P,U^{P},\delta \right)$ of projective modules and a morphism of weak chain U-complexes $f:(P,U^{P},\delta )\to (X,U^{X},d)$ which satisfies $f_{0}(U_{0}^{P})\subseteq U_{0}^{X}$, $f_{n}(U_{n}^{P})\subseteq U_{n}^{X}$ and $\delta_{n}(U_{n}^{P})\subseteq U_{n-1}^{P}$ for all n∈N.
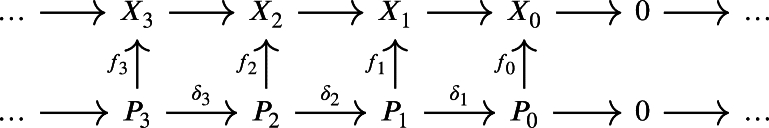

Notice that since $P_{0}\to X_{0}$ is surjective, then the morphism $g_{1}:Q_{1}\to X_{1}$ is also surjective, which implies $f_{1};P_{1}\to X_{1}$ is surjective. Similarly, we get $f_{n}$ is surjective for all n∈N.

We will show that $f_{n}(U_{n}^{P})=U_{n}^{X}$ for all n∈N. By definition, $U_{n}^{P}=f_{n}^{-1}(U_{n}^{X})\cap \delta_{n}^{-1}(U_{n-1}^{P})$. Therefore it is clear that $f_{n}(U_{n}^{P})\subseteq U_{n}^{X}$.

Now let $u_{n}\in U_{n}$. Since $f_{n}$ is surjective, then there exists $p_{n}\in P_{n}$ such that $f_{n}(p_{n})=u_{n}$. We need to show that $p_{n}\in \delta_{n}^{-1}(U_{n-1}^{P})$.

Notice that

$$\begin{array}{ccc} f_{n-1}\delta_{n}(p_{n})\; & =\; & d_{n}f_{n}(p_{n})\\ \; & =\; & d_{n}(u_{n})\in U_{n-1}^{X} \end{array}.$$

So, $\delta_{n}(p_{n})\in f_{n-1}^{-1}(U_{n-1}^{X})$. We also have that $\delta_{n-1}\delta_{n}(p_{n})\in U_{n-2}^{P}$, i.e., $\delta_{n}(p_{n})\in \delta_{n-1}^{-1}(U_{n-2}^{P})$. Therefore $\delta_{n}(p_{n})\in f_{n-1}^{-1}(U_{n-1}^{X})\cap \delta_{n-1}^{-1}(U_{n-2}^{P})=U_{n-1}^{P}$. We conclude that $p_{n}\in f_{n}^{-1}(U_{n}^{X})\cap \delta^{-1}(U_{n-1}^{P})=U_{n}^{P}$, and $f_{n}(U_{n}^{X})=U_{n}^{P}$.

We shall show that the morphism $f:(P,U^{P},\delta )\to (X,U^{X},d)$ induces an isomorphism H(f):H(P)→H(X). For the isomorphism between $H_{0}(P)$ with $H_{0}(X)$ we may use the same argument as in [10, Proposition 3.5.41]. Now for n∈N there exists a map

f‾n:Hn(P)=δn−1(Un−1P)Imδn+1+UnP→dn−1(Un−1X)Imdn+1+UnX=Hn(X)

First notice that Imδn=Imδn′ for all n∈N.

1. We will show that ${\bar{f}}_{n}$ is injective. Let yn+Imδn+1′+UnP∈Hn(P) with fn¯(yn+Imδn+1′+UnP)∈Imdn+1+UnX. Then
  $$f_{n}(y_{n})=d_{n+1}(x_{n+1})+u_{n}$$
  for some $x_{n+1}\in X_{n+1}$ and $u_{n}\in U_{n}^{X}$. Assume $u_{n}=f_{n}(v_{n})$ for some $v_{n}\in U_{n}^{P}$, hence we get
  $$f_{n}(y_{n}-v_{n})=d_{n+1}(x_{n+1}).$$
  Since $Q_{n+1}$ is the pullback of $P_{n+1}$ and $X_{n+1}$ to $X_{n}$, hence there exists $q_{n+1}\in Q_{n+1}$ such that
  $$\delta_{n+1}^{\prime }(q_{n+1})=y_{n}-v_{n}\;\;\text{and}\;\;g_{n+1}(q_{n+1})=x_{n+1}.$$
  Consequently, yn=δn+1′(qn+1)+vn∈Imδn+1′+UnP.
2. We will show that $\bar{f_{n}}$ is surjective. First we will show that there exists an isomorphism
  fn′‾:PnImδn+1′+UnP→XnImdn+1+UnX
  where fn′‾(yn+Imδn+1′+UnP)=fn(yn)+Imdn+1+UnX.
  First let $y_{n}=\delta_{n+1}^{\prime }(y_{n+1})+v_{n}$ for some $y_{n+1}\in Q_{n+1}$ and $v_{n}\in U_{n}^{P}$. It is clear that
  fn(yn)=fnδn+1′(yn+1)+fn(vn)=dn+1fn+1(yn+1)+fn(vn)∈Imdn+1+Un+1X
  Hence $\bar{f_{n}^{\prime }}$ is well-defined.
  Let xn+Imdn+1+UnX∈Xn/(Imdn+1+UnX). Then xn+Imdn+1∈Xn/Imdn+1≅Pn/Imδn+1 because of pullback. Hence there exists yn+Imδn+1+UnP such that fn′‾(yn+Imδn+1+UnP)=xn+Imdn+1+UnX.
  Now let yn+Imδn+1′+UnP∈PnImδn+1′+UnP such that fn(yn)∈Imdn+1+UnX. Then
  $$f_{n}(y_{n})=d_{n+1}(x_{n+1})+u_{n}$$
  for some $x_{n+1}\in X_{n+1}$ and $u_{n}\in U_{n}$. Hence for some $v_{n}\in U_{n}^{P}$,
  $$f_{n}(y_{n}-v_{n})=d_{n+1}(x_{n+1}),$$
  and since $Q_{n+1}$ is the pullback, then there exists $q_{n+1}\in Q_{n+1}$ such that
  $$\delta_{n+1}^{\prime }(q_{n+1})=y_{n}-v_{n}\;\;\text{and}\;\;f_{n+1}(q_{n+1})=x_{n+1}.$$
  Therefore, yn−vn∈Imδn+1′ and yn∈Imδn+1′+UnP. This concludes that $\bar{f_{n}^{\prime }}$ is an isomorphism. Now we have the following diagram
  δn−1(Un−1P)Imδn+1′+UnP↪PnImδn+1′+UnP≅dn−1(Un−1X)Imdn+1+UnX↪XnImdn+1+UnX
  Let zn+Imdn+1+UnX∈dn−1(Un−1X)+Imdn+1+UnX. By the above isomorphism, there exists yn+Imδn+1′+UnP∈PnImδn+1′+UnP such that
  fn(yn)+Imdn+1+UnX=zn+Imdn+1+UnX.
  We just need to show that $y_{n}\in \delta_{n}^{-1}(U_{n-1}^{P})$. Since $z_{n}\in d_{n}^{-1}(U_{n-1}^{X})$, then $d_{n}(z_{n})\in U_{n-1}^{X}$. So
  $$\begin{array}{ccc} z_{n}-f_{n}(y_{n})\; & =\; & d_{n+1}(x_{n+1})+u_{n}\;\text{for some}\;x_{n+1}\in X_{n+1}\;\text{and}\;u_{n}\in U_{n}^{X}\\ d_{n}(z_{n})-f_{n-1}\delta_{n}(y_{n})\; & =\; & d_{n}(z_{n}-f_{n}(y_{n}))\\ \; & =\; & d_{n}d_{n+1}(x_{n+1})+d_{n}(u_{n})\in U_{n-1}^{X} \end{array}$$
  Therefore, $f_{n-1}\delta_{n}(y_{n})\in U_{n-1}^{X}$ and this implies $\delta_{n}(y_{n})\in U_{n-1}^{P}$

We conclude that the natural functor

$$K_{U}^{-}(R-Proj)\to D_{U}^{-}(R)$$

is dense. By the same argument as in [10, Lemma 3.5.41] we conclude that the functor is fully faithful. □

## 5 Correspondences between weak chain U-complexes and chain complexes

We have constructed the homotopy category and the derived category of weak chain U-complexes from the weak chain U-complexes. This section will discuss some correspondences between these categories and the usual chain complexes categories. We shall show that there is a pair of adjoint functors between $C_{U}(R)$ and C(R). Then, we show that these functors induce functors between homotopy categories and between derived categories.

### 5.1 A functor from $C_{U}(R)$ to C(R)

For every object $\left(X_{n},U_{n},d_{n}\right)$ in $C_{U}(R)$, we can construct a chain complex $\left(\frac{X_{n}}{U_{n}},\bar{d_{n}}\right)$ with

$$\bar{d_{n}}:\frac{X_{n}}{U_{n}}\to \frac{X_{n-1}}{U_{n-1}},\;\bar{d_{n}}(x+U_{n})=d_{n}(x)+U_{n-1}.$$

We want to verify whether $\bar{d_{n}}$ is a well-defined map. For that, we have the following lemma.

Lemma 5.1 *Let* M,N *be R-modules with* U⊆M *and* V⊆N *their submodules. If* f:M→N *is an R-module homomorphism with* f(U)⊆V *, then there exists an R-module homomorphism* $\bar{f}:\frac{M}{U}\to \frac{N}{V}$ *with*

$$\bar{f}:m+U\mapsto f(m)+V$$

Motivated by the previous lemma, we can try to make a correspondence between objects in $C_{U}(R)$ and C(R).

Theorem 5.2 *The chain complex* $\left(\frac{X_{n}}{U_{n}},\bar{d_{n}}\right)$ *is an object in* C(R) *and there is a functor* $F:C_{U}(R)\to C(R)$ *with*

$$F((X_{n},U_{n},d_{n}))=\left(\frac{X_{n}}{U_{n}},\bar{d_{n}}\right).$$

Proof The first part is simple. Let $x+U_{n+1}\in X_{n+1}/U_{n+1}$. We have

$$\bar{d_{n}}\;\bar{d_{n+1}}(x+U_{n+1})=\bar{d_{n}}(d_{n+1}(x)+U_{n})=d_{n}d_{n+1}(x)+U_{n-1}=0+U_{n-1}$$

since $d_{n}d_{n+1}(x)\in U_{n-1}$.

Now, we will show that the correspondence above is functorial. Indeed, if we have a morphism f:X→Y of weak chain U-complexes $\left(X_{n},U_{n}^{X},d_{n}^{X}\right)$ to $\left(Y_{n},U_{n}^{Y},d_{n}^{Y}\right)$, it induces the sequence of morphisms

$$\bar{f}:=\left(\bar{f_{n}}:\frac{X_{n}}{U_{n}^{X}}\to \frac{Y_{n}}{U_{n}^{Y}}\right)$$

with $\bar{f_{n}}:x_{n}+U_{n}^{X}\mapsto f(x_{n})+U_{n}^{Y}$. Verifying that it is a morphism of chain complexes is simple. Notice that

$$\bar{f_{n-1}}\;\bar{d_{n}^{X}}(x_{n}+U_{n})=\bar{f_{n-1}}(d_{n}^{X}(x_{n})+U_{n-1}^{X})=f_{n-1}d_{n}^{X}(x_{n})+U_{n-1}^{Y}=d_{n}^{Y}f_{n}(x_{n})+U_{n-1}^{Y}=\bar{d_{n}^{Y}}(f_{n}(x_{n})+U_{n}^{Y})=\bar{d_{n}^{Y}}\;\bar{f_{n}}(x_{n}+U_{n}^{X}).$$

So, we have $\bar{f_{n-1}}\;\bar{d_{n}^{X}}=\bar{d_{n}^{Y}}\;\bar{f_{n}}$. It's not hard to see that the above map preserves the composition of morphism and identity. □

### 5.2 A functor from C(R) to $C_{U}(R)$

For any $\left(X_{n},d_{n}\right)$ in Ob(C(R)), we have several natural correspondence to an object in $C_{U}(R)$. Some of the natural constructions are as follows:

1. $\left(X_{n},0,d_{n}\right)$
2. $\left(X_{n},X_{n},d_{n}\right)$
3. (Xn,Imdn+1,dn)
4. $\left(X_{n},\mathrm{Ker}\;d_{n},d_{n}\right)$

Now, if we have $f=(f_{n}:X_{n}\to Y_{n})\in {\mathrm{Hom}}_{C(R)}(X,Y)$, by definition it's obvious that $f_{n}(0)=0$ and $f_{n}(X_{n})\subseteq Y_{n}$. Now, notice that if $y_{n}=d_{n+1}^{X}(x_{n+1})$, we have

fn(yn)=fndn+1X(xn+1)=dn+1Yfn+1(xn+1)∈Imdn+1Y

Hence, we have fn(Imdn+1X)⊆Imdn+1Y. On the other hand, if $d_{n}^{X}(x_{n})=0$, we then have

$$d_{n}^{Y}f_{n}(x_{n})=f_{n-1}d_{n}^{X}(x_{n})=f_{n-1}(0)=0$$

so that $f_{n}(\mathrm{Ker}\;d_{n}^{X})\subseteq \mathrm{Ker}\;d_{n}^{Y}$. From these observations, we have all four of our constructions, each defining a functor from C(R) to $C_{U}(R)$.

Now, let $G:C(R)\to C_{U}(R)$ denote our first functor; i.e., we have

$$G((X_{n},d_{n}))=(X_{n},0,d_{n})$$

Theorem 5.3 *The functor* $F:C_{U}(R)\to C(R)$ *defined in the previous section is left-adjoint to G.*

Proof Let $X\in C_{U}(R)$ and Y∈C(R). We need to show that there is a natural bijection

$$\phi :{\mathrm{Hom}}_{C(R)}(F(X),Y)\to {\mathrm{Hom}}_{C_{U}(R)}(X,G(Y)).$$

Now, let

$$f:F(X)=\left(\frac{X_{n}}{U_{n}},\bar{d_{n}}\right)\to Y$$

be a morphism of chain complexes with $f_{n}:\frac{X_{n}}{U_{n}}\to Y_{n}$. Naturally, there exists $g:X=(X_{n},U_{n},d_{n}^{X})\to G(Y)=(Y_{n},0,d_{n}^{Y})$ such that $g_{n}:X_{n}\to Y_{n}$ is a composition of canonical projection $X_{n}↠\frac{X_{n}}{U_{n}}$ with $f_{n}$. Since $g_{n}$ maps $U_{n}$ to 0 and

$$g_{n-1}d_{n}^{X}(x_{n})=f_{n-1}(d_{n}^{X}(x_{n})+U_{n-1})=f_{n-1}\bar{d_{n}^{X}}(x_{n}+U_{n})=d_{n}^{Y}f_{n}(x_{n}+U_{n})=d_{n}^{Y}g_{n}(x_{n})$$

we have that *g* is a morphism of weak chain U-complex. From this naturality, we may define ϕ(f)=g.

On the other hand, let

$$g:X=(X_{n},U_{n},d_{n}^{X})\to G(Y)=(Y_{n},0,d_{n}^{Y})$$

with $g_{n}:X_{n}\to Y_{n}$ is a morphism of weak chain U-complex. It is clear that $g_{n}$ maps $U_{n}$ to 0. Therefore, the map $f_{n}:\frac{X_{n}}{U_{n}}\to Y_{n}$ given by $f_{n}(x+U_{n})=g(x)$ is well-defined. It is not difficult to check that $f=(f_{n}:\frac{X_{n}}{U_{n}}\to Y_{n})$ is a morphism of weak chain U-complex and that $g_{n}$ is a composition of the canonical projection $X_{n}↠\frac{X_{n}}{U_{n}}$ with $f_{n}$.

Since

$$f_{n-1}\bar{d_{n}^{X}}(x_{n}+U_{n})=f_{n-1}(d_{n}^{X}(x_{n})+U_{n-1})=g_{n-1}d_{n}^{X}(x_{n})=d_{n}^{Y}g_{n}(x_{n})=d_{n}^{Y}f_{n}(x_{n}+U_{n})$$

we have that *f* is a morphism of chain complexes. We then define ψ(g)=f. It's easy to see that *ψ* is the inverse of *ϕ* (and vice-versa). □

Remark 5.4

1. The unit $\eta :{\mathrm{id}}_{C_{U}(R)}\to G\circ F$ from the adjunction above is given by
  $$\eta_{X}:X\to (G\circ F)(X)$$
  with
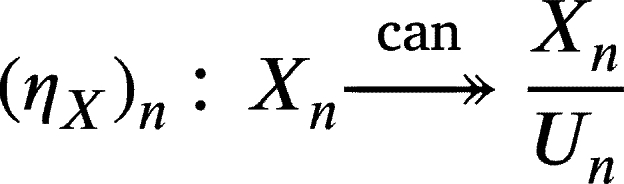
 Dually, the counit $\varepsilon :F\circ G\to {\mathrm{id}}_{C(R)}$ is given by
  $$\varepsilon_{Y}:(F\circ G)(Y)\to Y$$
  with
  $${\left(\varepsilon_{Y}\right)}_{n}={\mathrm{id}}_{Y_{n}}:Y_{n}\to Y_{n}$$
2. The counit of the adjunction above defines a natural isomorphism from (F∘G)(Y) to *Y*. As a consequence, *G* is a fully-faithful functor. Using this functor, we can see C(R) as a full subcategory of $C_{U}(R)$.

### 5.3 The homology functor

In this section, we are interested in investigating the connection between the homology functor defined in C(R) and the homology functor defined in $C_{U}(R)$.

Let $X=(X_{n},U_{n},d_{n})$ be a weak chain U-complex. Notice that by definition we have $U_{n}$ and Imdn+1 are submodules of $d_{n}^{-1}(U_{n-1})$. We have defined the *n*-th homology module of *X* as

Hn(X)=dn−1(Un−1)Un+Imdn+1

Now, apply the functor *F* to *X*, we have a chain complex $F(X)=\left(\frac{X_{n}}{U_{n}},\bar{d_{n}}\right)$. This complex can be identified with a weak chain U-complex $(G\circ F)(X)=\left(\frac{X_{n}}{U_{n}},0,\bar{d_{n}}\right)$. Taking the homology of (G∘F)(X), we have

Hn((G∘F)(X))=dn−1(0)0+Imdn+1=KerdnImdn+1

One might notice that the expression on the right-hand side is a homology module of a chain complex *X*. In other word, the homology functor of chain complex $H_{n}:C(R)\to R-Mod$ is naturally isomorphic to the composition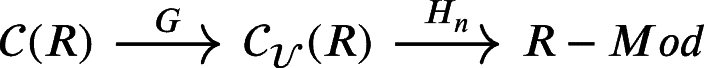 Therefore, we have the following commutative diagram:
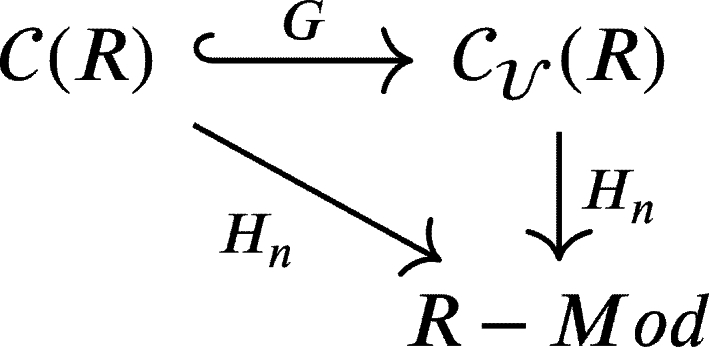

Theorem 5.5 *The counit of the adjunction* (F,G) *gives a quasi-isomorphism* $\eta_{X}:X\to (G\circ F)(X)$ *.*

Proof Notice that

$$\bar{d_{n}^{-1}}(0)=\left\{x_{n}+U_{n}\in \frac{X_{n}}{U_{n}}:\bar{d_{n}}(x_{n}+U_{n})=0\right\}=\left\{x_{n}+U_{n}\in \frac{X_{n}}{U_{n}}:d_{n}(x_{n})+U_{n-1}=0\right\}=\left\{x_{n}+U_{n}\in \frac{X_{n}}{U_{n}}:d_{n}(x_{n})\in U_{n-1}\right\}=\left\{x_{n}+U_{n}\in \frac{X_{n}}{U_{n}}:x_{n}\in d_{n}^{-1}(U_{n-1})\right\}=\frac{d_{n}^{-1}(U_{n-1})}{U_{n}},$$

and

Im(dn+1‾)={dn+1‾(xn+1+Un+1):xn+1+Un+1∈Xn+1Un+1}={dn+1(xn+1)+Un:xn+1∈Xn+1}={yn+Un:yn∈Im(dn+1)}=Un+Im(dn+1)Un.

Since Un⊆Un+Im(dn+1)⊆dn−1(Un−1), by the third isomorphism theorem for modules, we have the following isomorphism:
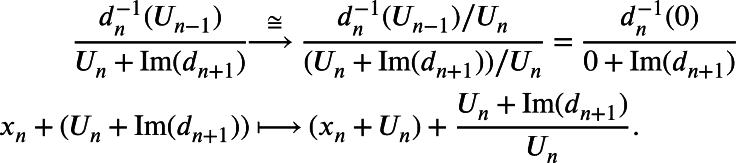

Since the counit $\eta_{X}:X\to (G\circ F)(X)$ is defined as

$${\left(\eta_{X}\right)}_{n}:X_{n}\to \frac{X_{n}}{U_{n}},\;{\left(\eta_{X}\right)}_{n}(x_{n})=x_{n}+U_{n},$$

the above isomorphism is no other than $H_{n}(\eta_{X})$. □

We can rewrite the above theorem in another way. Suppose that we have two homology functors defined on $C_{U}(R)$ with the first one as defined above and the second one defined via the homology functor of C(R) as a composition.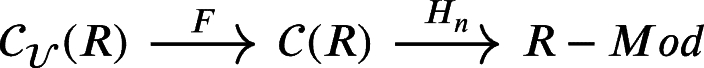 The theorem above says that those functors are naturally isomorphic, resulting in the following commutative diagram:
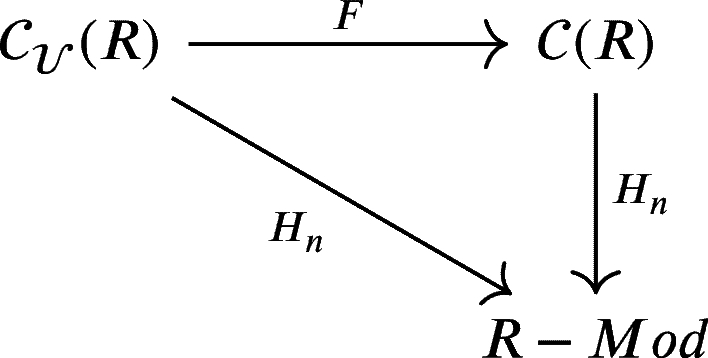


### 5.4 Derived equivalence

Theorem 5.6 *The functor* $F:C_{U}(R)\to C(R)$ *and* $G:C(R)\to C_{U}(R)$ *preserves the homotopy relation. In other words:*

1. *If* f∼g *in* $C_{U}(R)$ *, then* F(f)∼F(g) *in* C(R) *.*
2. *If* f∼g *in* C(R) *, then* G(f)∼G(g) *in* $C_{U}(R)$ *.*

Proof We prove each part separately.

1. Let f,g be a morphism of weak chain U-complexes such that f∼g and $k_{n}:X_{n}\to Y_{n+1}$ defines a homotopy from *f* to *g*. Since $k_{n}(U_{n}^{X})\subseteq U_{n+1}^{Y}$, we have an induced sequence $\bar{k}=(\bar{k_{n}})$ given by
  $$\bar{k_{n}}:\frac{X_{n}}{U_{n}^{X}}\to \frac{Y_{n+1}}{U_{n+1}^{Y}},\;\bar{k_{n}}(x_{n}+U_{n}^{X})=k_{n}(x_{n})+U_{n+1}^{Y}.$$
  Now, notice that for $x_{n}+U_{n}^{X}\in X_{n}/U_{n}^{X}$ we have
  $$F(f)(x_{n}+U_{n}^{X})-F(g)(x_{n}+U_{n}^{X})=(f(x_{n})+U_{n}^{Y})-(g(x_{n})+U_{n}^{Y})=(f(x_{n})-g(x_{n}))+U_{n}^{Y}=(k_{n-1}d_{n}^{X}(x_{n})+d_{n+1}^{Y}k_{n}(x_{n}))+U_{n}^{Y}=(k_{n-1}d_{n}^{X}(x_{n})+U_{n}^{Y})+(d_{n+1}^{Y}k_{n}(x_{n})+U_{n}^{Y})=\bar{k_{n-1}}(d_{n}^{X}(x_{n})+U_{n-1}^{X})+\bar{d_{n+1}^{Y}}(k_{n}(x_{n})+U_{n+1}^{Y})=\bar{k_{n-1}}\;\bar{d_{n}^{X}}(x_{n}+U_{n}^{X})+\bar{d_{n+1}^{Y}}\;\bar{k_{n}}(x_{n}+U_{n}^{X}).$$
  Thus, we have
  $$F(f)-F(g)=\bar{k_{n-1}}\;\bar{d_{n}^{X}}+\bar{d_{n+1}^{Y}}\;\bar{k_{n}}$$
  so F(f)∼F(g) with $\bar{k}=(\bar{k_{n}})$ as the homotopy between them.
2. Let f,g be a morphism of chain complex such that f∼g with homotopy $k=(k_{n})$ between them. Then, we also have that *k* is a homotopy between G(f) and G(g) of weak chain U-complexes. □

Due to the above theorem, we now have a correspondence between the homotopy category of chain complexes and the homotopy category of weak chain U-complexes.

Corollary 5.7 *The functor F and G induce functors* $K(F):K_{U}(R)\to K(R)$ *and* $K(G):K(R)\to K_{U}(R)$ *such that the following squares commute:*

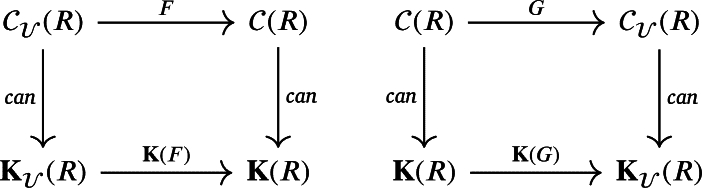

**Notes**: In the above diagram, the vertical maps are the canonical maps between the category of chain complexes (weak chain U-complexes) and its homotopy category.

Proof We only need to show that this is true for the first diagram. First, consider the following composition of functors.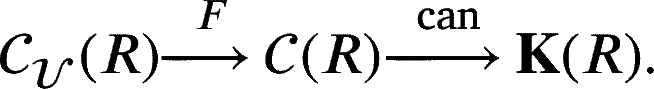 The composition above maps the nullhomotopic morphism to the zero morphism of K(R). Thus, we can factorize the composition via $K_{U}(R)$. In other words, we get the first commutative square. □

Theorem 5.8 *The functors* $F:C_{U}(R)\to C(R)$ *and* $G:C(R)\to C_{U}(R)$ *preserve quasi-isomorphism.*

Proof We need to show two things. First, for any quasi-isomorphism *f* in $C_{U}(R)$, F(f) is a quasi-isomorphism in C(R). Similarly, we also need to show that for any quasi-isomorphism *f* in C(R), we have G(f) a quasi-isomorphism in $C_{U}(R)$.

1. Let f:X→Y be a quasi-isomorphism in $C_{U}(R)$. Now, observe the following commutative square:
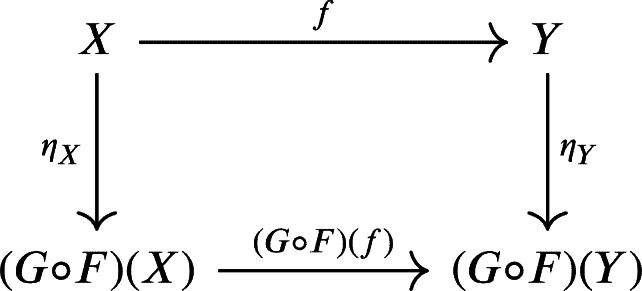
 Since $\eta_{X}$ and $\eta_{Y}$ defines a quasi-isomorphism, by applying the homology functor $H_{n}$ on the commutative square above, we have
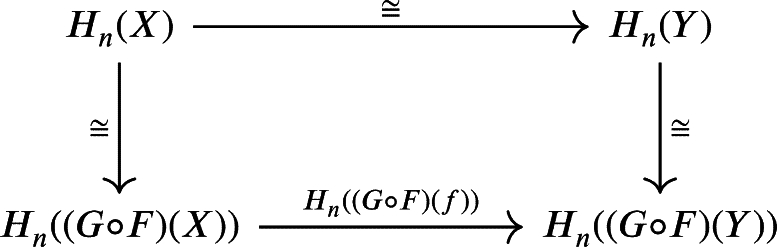
 Hence, $H_{n}((G\circ F)(f))$ is an isomorphism for every n∈Z. In other word, (G∘F)(f) is a quasi-isomorphism between (G∘F)(X) and (G∘F)(Y). We can then conclude that F(f):F(X)→F(Y) is a quasi-isomorphism.
2. Let f:X→Y be a quasi-isomorphism in C(R). Since there exists a natural isomorphism $H_{n}\circ G≅H_{n}$ (with the left one is a homology functor in $C_{U}(R)$ and the right one is a homology functor in C(R)), we have G(f):G(X)→G(Y) a quasi-isomorphism. □

Since the functor *F* and *G* define a functor between its homotopy category and it preserves the quasi-isomorphism, we can now talk about the induced functor between the derived category of chain complexes and weak chain U-complex.

Corollary 5.9 *Functor F and G induce functors* $D(F):D_{U}(R)\to D(R)$ *and* $D(G):D(R)\to D_{U}(R)$ *which yield the following commutative squares:*

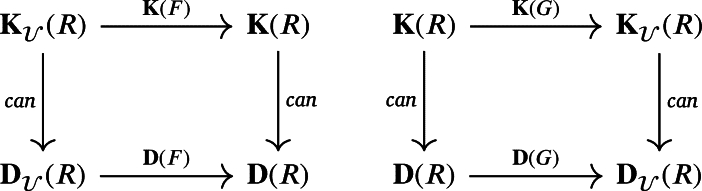

Proof We only prove the first diagram; the second one follows similarly. Observe the composition of functors.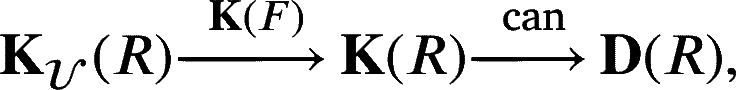 which sends the quasi-isomorphism in $K_{U}(R)$ into an isomorphism in D(R). Thus, we can factorize the above composition via $D_{U}(R)$, yielding the first commutative square. □

The previous results suggest a conjecture.

Conjecture 5.10 *The functor* $D(F):D_{U}(R)\to D(R)$ *defines an equivalence of triangulated category with* $D(G):D(R)\to D_{U}(R)$ *as its (pseudo-)inverse.*

## CRediT authorship contribution statement

**Fajar Yuliawan:** Validation, Supervision, Project administration, Methodology, Investigation, Formal analysis, Conceptualization. **Intan Muchtadi-Alamsyah:** Writing – review & editing, Writing – original draft, Supervision, Project administration, Methodology, Investigation, Funding acquisition, Formal analysis, Conceptualization. **Valerian Pratama:** Writing – review & editing, Writing – original draft, Visualization, Software, Methodology, Investigation, Formal analysis. **Gustina Elfiyanti:** Writing – review & editing, Writing – original draft, Validation, Methodology, Investigation, Formal analysis, Conceptualization.

## Declaration of Competing Interest

The authors declare that they have no known competing financial interests or personal relationships that could have appeared to influence the work reported in this paper.

## Data Availability

Data regarding this research is included in the manuscript.
